# Analysis of CRISPR gene drive design in budding yeast

**DOI:** 10.1099/acmi.0.000059

**Published:** 2019-09-11

**Authors:** Yao Yan, Gregory C. Finnigan

**Affiliations:** ^1^​ Department of Biochemistry and Molecular Biophysics, Kansas State University, 141 Chalmers Hall, Manhattan, KS 66506, USA

## Abstract

Control of biological populations remains a critical goal to address the challenges facing ecosystems and agriculture and those posed by human disease, including pests, parasites, pathogens and invasive species. A particular architecture of the CRISPR/Cas biotechnology – a gene drive – has the potential to modify or eliminate populations on a massive scale. Super-Mendelian inheritance has now been demonstrated in both fungi and metazoans, including disease vectors such as mosquitoes. Studies in yeast and fly model systems have developed a number of molecular safeguards to increase biosafety and control over drive systems *in vivo*, including titration of nuclease activity, anti-CRISPR-dependent inhibition and use of non-native DNA target sites. We have developed a CRISPR/Cas9 gene drive in *Saccharomyces cerevisiae* that allows for the safe and rapid examination of alternative drive designs and control mechanisms. In this study, we tested whether non-homologous end-joining (NHEJ) had occurred within diploid cells displaying a loss of the target allele following drive activation and did not detect any instances of NHEJ within multiple sampled populations. We also demonstrated successful multiplexing using two additional non-native target sequences. Furthermore, we extended our analysis of ‘resistant’ clones that still harboured both the drive and target selection markers following expression of *Streptococcus pyogenes* Cas9; *de novo* mutation or NHEJ-based repair could not explain the majority of these heterozygous clones. Finally, we developed a second-generation gene drive in yeast with a guide RNA cassette integrated within the drive locus with a near 100 % success rate; resistant clones in this system could also be reactivated during a second round of Cas9 induction.

## Introduction

The use of CRISPR/Cas genomic editing has allowed for recent advances across many fields, including agriculture, biotechnology and basic laboratory research [1–3]. The introduction of targeted chromosomal breaks within a genome of interest coupled with DNA repair allows for the generation of nearly any conceivable genetic modification [4, 5]. One powerful arrangement that utilizes CRISPR/Cas has the ability to rapidly ‘force’ a genetic element of choice through a native population – a gene drive (GD) system [6–8]. In theory, this biotechnology could be used to either deliver a desired trait to a population or eliminate native populations (for example, through extreme bias of sex determination). The control of specific biological populations is a critical challenge facing numerous industries and is a problem posed by global health epidemics, including animal and plant pests and parasites, the spread of pathogens (via insect vectors) and the alteration of native environments by invasive organisms [9, 10]. Given the widespread future applicability of CRISPR GDs, additional laboratory study of biosafety [6, 11], control [12–15] and reversal systems [16] is critical.

Current CRISPR-based GDs have been developed and tested under laboratory conditions in fungi [12, 13, 17–19], insects [7, 8, 20, 21], and even vertebrates [22], with varying success rates. Our previous work in budding yeast developed a highly tractable drive system that can be used as a platform for the testing of novel drive arrangements, CRISPR components, DNA repair and modes of inhibition or control [12, 13, 17, 23, 24]. The basic mechanism of a GD includes the integration of the basic CRISPR system (nuclease and corresponding guide RNA expression cassettes) at a particular genomic locus. Expression of Cas9/sgRNA within a diploid genome allows for the introduction of a double-strand break (DSB) in the target allele within the same locus where the GD was positioned on the homologous chromosome. The DSB will be repaired using homology-directed repair (HDR) and the drive-containing chromosome as the source of donor DNA; the GD cassette is then copied to replace the entire target locus. The heterozygous pairing of a drive-containing individual with a wild-type (WT) individual (heterozygote) yields all *homozygous* diploid progeny for the GD; this allows for *super*-Mendelian inheritance of the drive locus through a population.

To date, some of our findings in *Saccharomyces cerevisiae* have also been tested in higher eukaryotes, including targeting of non-native DNA sequences within target loci (such as eGFP or other programmed artificial sequences) [17, 25, 26], titration of Cas9 activity within a GD [13, 15] and split drives that separate guide RNAs from nucleases across multiple loci [14, 24]. A number of important ongoing questions remain open for GD research, including the occurrence of drive resistance; mechanisms to slow, inhibit, or reverse active drives; and how population control may impact larger ecosystems during possible field deployment of drive systems. In this study, we examine our original first-generation CRISPR system in budding yeast and test (i) the possible occurrence of DNA repair by the non-homologous end-joining (NHEJ) system; (ii) additional controls for our examination of the drive and target loci following drive action; (iii) multiplexing to independent target sequences; (iv) examination of ‘resistant’ clone formation; and (v) a modified second-generation drive harbouring an integrated guide RNA cassette.

## Methods

### Yeast strains and plasmids

The budding yeast (*S. cerevisiae*) strains used are presented in Table S1 (available with the online version of this article) (also see Fig. S1). Molecular techniques were used for the generation of all artificial constructs [27]. Prior to genomic integration, engineered DNA assemblies were first created on *CEN*-based plasmids using *in vivo* ligation and homologous recombination [28]. Plasmids were confirmed with diagnostic PCRs and DNA sequencing. Amplified PCRs of assembled expression cassettes (from isolated chromosomal DNA, generated plasmids, or synthetic genes as templates) were transformed into cells using a lithium acetate-based protocol and integrated at the *HIS3* locus, often in multiple overlapping fragments using standard selection markers (*SpHIS5* or Kan^R^). Chromosomal insertions were confirmed by both PCRs and DNA sequencing. The plasmids used in this study are listed in Table S2 (see Fig. S1 for sequences). The expression cassettes for *Streptococcus pyogenes* guide RNAs were based on previous work [29]. Briefly, these included the yeast *SNR52* promoter, a 20 bp variable crRNA sequence, a 79 bp tracrRNA sequence and the *SUP4* terminator (Fig. S1). Custom genes were synthetized (Genscript) into a pUC57-Kan^R^ vector and sub-cloned to pRS425 or pRS426 using two unique flanking restriction sites.

### Culture conditions

Yeast were propagated on solid agar plates or in liquid media cultures. Rich medium included 2 % peptone, 1 % yeast extract and 2 % dextrose. Synthetic drop-out media included yeast nitrogen base, ammonium sulfate, amino acids and a carbon source. For preinduction, a mixture of raffinose (2 %) and sucrose (0.2 %) was used for overnight cultures. For galactose activation (2 %), rich medium (YP) was used. Sugars were not autoclaved and instead were filter-sterilized. Synthetic drop-out medium also contained added tryptophan (not autoclaved). A concentration of 240 µg ml^−1^ was used for G418-containing plates. Yeast strains were all maintained at 30 °C on plates or in liquid culture for the indicated times.

### CRISPR editing and gene drives

CRISPR editing of haploid yeast included culturing GFY-2383 yeast in a preinduction medium overnight, followed by back-dilution into rich medium containing galactose for 5 h. Cells were harvested, transformed with the sgRNA(u2) (pGF-V809) high-copy plasmid, recovered overnight in rich medium containing galactose, and plated onto SD-LEU medium for 3–4 days. Clonal isolates were tested by growth (loss of G418 resistance), chromosomal DNA extraction, PCR and DNA sequencing. First, for the creation and activation of CRISPR GDs, haploid strains (harbouring Cas9) were transformed with the sgRNA-containing plasmid(s), if necessary. Second, haploids were mated to the haploid target strain of the opposite mating type for 20–24 h on rich medium. Third, diploids were selected on SD-LEU-HIS, SD-LEU-URA-HIS, or SD-URA-HIS for three consecutive rounds (24–48 h incubation times). Fourth, cells were cultured in preinduction medium overnight: S+Raff/Suc-LEU, S+Raff/Suc-LEU-URA, or S+Raff/Suc-URA-HIS and S+Raff/Suc-URA-LEU-HIS. Fifth, strains were transferred into rich medium containing galactose (typically for 5 h). Sixth, cells were harvested, diluted in sterile water to approximately 200–500 cells ml^−1^ and plated onto SD-LEU, SD-LEU-URA, or SD-URA for 48 h. Finally, yeast colonies were transferred (sterile velvet) to additional plates, such as SD-HIS or SD-URA-HIS for 20–24 h before imaging. Clonal isolates were sampled at random from the final growth plates in at least triplicate. The safety mechanisms in place included a number of features such as targeting of *S. pyogenes* Cas9 to sequences not found within the native yeast genome (*SpHIS5*, mCherry, u1′, u2) [25], control of Cas9 expression under a galactose-responsive promoter (and use of dextrose in all plate types to repress nuclease expression), use of unstable high-copy plasmids [6] to harbour the sgRNA cassettes and programmed self-excision (u2) or (u2′) sites flanking all drive modules [13]. The diploid yeast strains generated prior to and after drive activation (or mock activation) were all destroyed following experimentation (only original haploids were preserved). Of note, our modified GD system (GD2, strains GFY-4325/4226) contained an integrated sgRNA cassette proximal to the Cas9 gene. However, all other safety features still applied, including the choice of (u1) as the crRNA sequence included within the drive.

## Results

### Additional controls for a first-generation CRISPR GD system

**Fig. 1.**
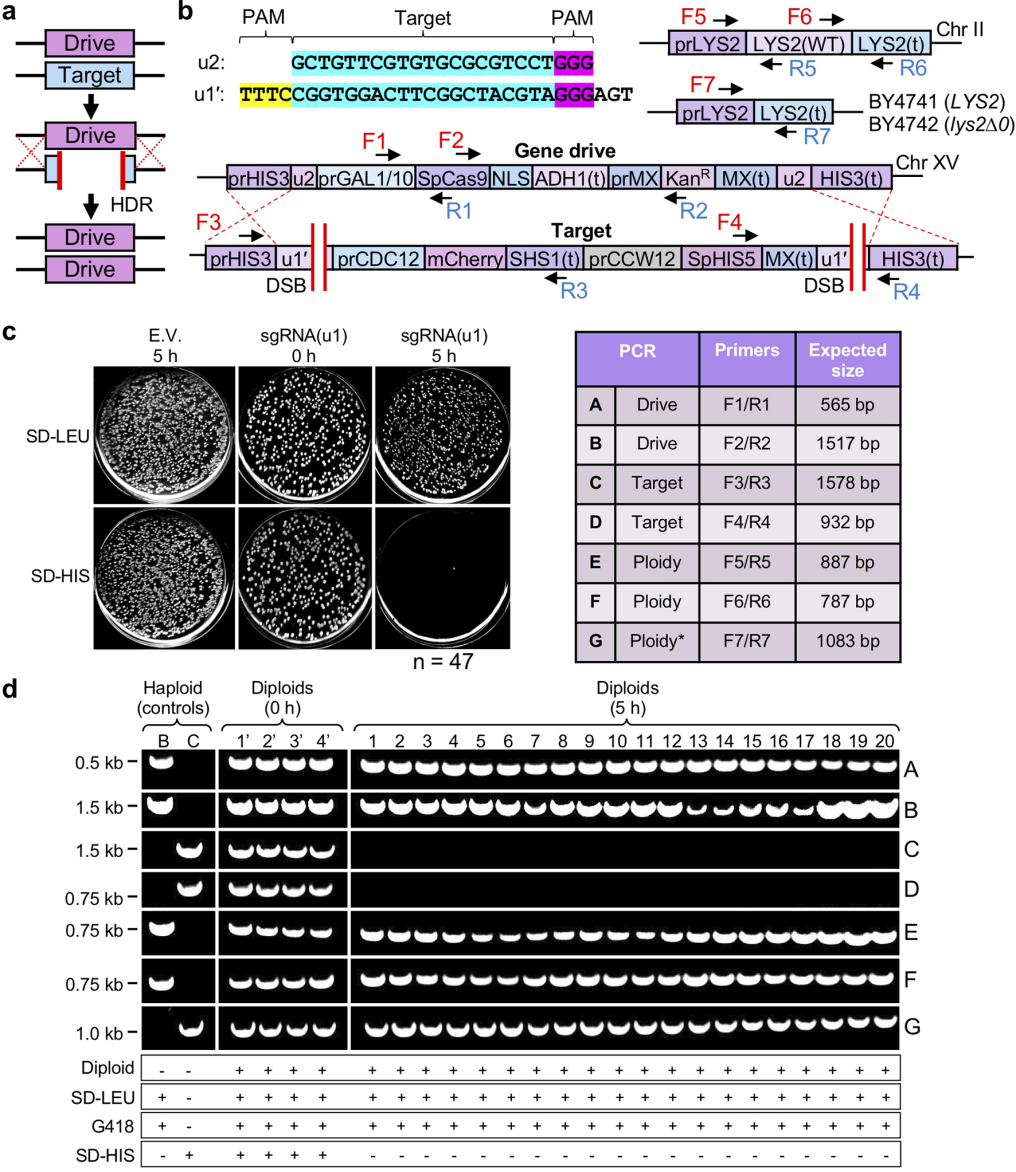
A first-generation CRISPR GD in budding yeast. (a) Mode of action for an artificial GD. Pairing between drive and target (typically WT) individuals and activation of the drive system results in cleavage of the target allele, copying of the drive cassette through homology-directed repair and conversion to the homozygous condition. (b) Design of an artificial drive/target system in *S. cerevisiae*. Unique CRISPR target sites (u1’ and u2) were introduced flanking both the drive and target cassettes at the yeast *HIS3* locus. The *GAL1/10* promoter is repressed when cells are exposed to dextrose and activated in medium containing galactose. The *CCW12* promoter (cell wall component) provides constitutive expression of the target selection marker – *S. pombe HIS5* (functional equivalent of *S. cerevisiae HIS3* that allows for growth on medium lacking histidine). A codon-optimized *S. pyogenes* Cas9 contains a C-terminal SV40 NLS. Haploid yeast strains contain unique alleles for *LYS2* (BY4741, GD strain, *LYS2*; BY4742, target strain, *lys2*∆0). The sgRNA(u1) expression cassette is present on a high-copy *LEU2*-based plasmid (not illustrated). (c) Left: following treatment with galactose (0 or 5 h), strains were plated onto permissive medium (SD-LEU) followed by transfer to both SD-LEU and SD-HIS plates (examples shown) before imaging. EV, empty pRS425 vector. The GD condition (far right) was tested in 47 independent diploid strains (see Fig. S2). Separate plate images (entire plate) were edited for contrast and clarity. (d) Diagnostic PCRs of clonal yeast isolates from (c) following GD activation and resistance to G418. Top right: table of oligonucleotides used and expected DNA fragment sizes (bp). Haploid controls included strains GFY-2383 (drive, control B) and GFY-3733 (target, control C). Asterisk, the expected PCR size for PCR-G was 5573 bp for control B and 1083 bp for control C. Horizontal white lines designate separate DNA gels; vertical white lines have been included for clarity. Determination of diploid status also utilized mating assays to control strains (Fig. S3) and amplification of the *LYS2* (PCRs E-G) and *MET15* loci (Fig. S4). Unmodified DNA gels are presented in Fig. S7.

The intended mechanism of an artificial GD includes DSB formation within the target (often multiplexing to more than one cut site), excision of the target DNA and replacement of the entire locus using the drive-containing chromosome as the source of donor material and HDR (Fig. 1a). This system requires a polyploid (diploid) genome in order to convert heterozygous cells to the homozygous state for the GD locus. Therefore, we re-examined our first-generation GD system (GD1) [13] with several modifications and additional experimental controls (Fig. 1b). First, we developed a new target haploid strain that included identical flanking (u1) sequences [25] distinct from the yeast genome. The new version (u1′) included the same target sequence and PAM for use with *S. pyogenes* Cas9, and also a 5′ PAM sequence (5′-TTTV-3′) for the *Francisella novicida* Cas12a nuclease [30], as well as one possible PAM sequence for *Staphylococcus aureus* Cas9 (5′-NNGRRT-3′) [31] for future studies that could all utilize an overlapping core target sequence motif (Fig. 1b).

Activation of our GD1 system required the treatment of diploid cells (pairing between the two yeast mating types, one harbouring the GD and the other harbouring the target locus) with galactose for the induction of Cas9 expression. The sgRNA(u1) expression cassette was included on a high-copy plasmid. Success of the GD system included testing colonies on synthetic medium lacking histidine. The target locus included constitutive expression of *Schizosaccharomyces pombe HIS5* (the functional equivalent of *S. cerevisiae HIS3*) that allowed for growth on SD-HIS plates; copying of the drive allele to replace the target removed the selection marker and caused sensitivity to this condition. The inclusion of an empty vector control (no guide RNA) resulted in no loss of growth on SD-HIS (Fig. 1c, left). Following preinduction (0 h, raffinose/sucrose mixture), only a small number of colonies were sensitive (Fig. 1c, middle). However, after 5 h galactose treatment, nearly every yeast colony was inviable on SD-HIS plates across numerous independent trials (Fig. 1c, right*,* Fig. S2). Subsequent analyses of individual diploid clones were performed to test whether the target locus was removed as expected (Fig. 1d). Using diagnostic PCRs (oligonucleotides found in Table S3) for unique DNA elements within the drive or target loci, we tested 4 randomly chosen isolates from the 0 h condition versus 20 isolates for the 5 h condition that all maintained growth on plates containing G418 due to the presence of the Kan^R^ selection marker present within the drive locus (PCRs A-D). Importantly, we included the two original haploid strains (drive and target) as further controls for this analysis. In all clones sampled after the expression of Cas9, the drive locus was present (PCRs A,B), whereas the target locus could not be amplified (PCRs C,D) compared to the 0 h control and the original haploid strain.

We chose clonal isolates that were diploids using two independent assays for subsequent analyses. Examination of GD success requires conversion of the heterozygous diploid genome to the homozygous state (copying of the drive and loss of the target allele). Selected clones (that also maintained G418 resistance) were mated against known haploid strains of the two mating types and tested for the presence of two selectable markers (one from each haploid). Only strains that were able to mate to form diploids and contain both markers would survive under dual selection; diploid strains would be unable to mate with haploid controls and would be sensitive to the final selection challenge (Fig. S3). Clones that failed to mate in these initial assays (diploids) were chosen for further analysis by PCR. Next, we included a set of diagnostic PCRs to analyse the diploid genome. The BY4741 and BY4742 laboratory strains include distinct alleles of the *LYS2* and *MET15* markers (Table S1 and Fig. S1). Two regions of the *LYS2* coding sequence (PCRs E,F) were amplified to test for the presence of the *LYS2* gene (from BY4741, haploid drive genome) and one PCR was performed (PCR G) using primers flanking the *LYS2* coding sequence to test for the *lys2∆0* allele (from BY4742, haploid target genome). While haploid controls only demonstrated the inclusion of one of the two possible *LYS2* alleles, the diploids from our GD experiments (first confirmed through the mating test) included amplification of both *LYS2* and *lys2∆0* alleles (Fig. 1d). Finally, as an independent test of a different chromosome not containing *LYS2*, we examined 10 isolates by PCR at the *MET15* locus (Fig. S4). In this case, the BY4741 genome (drive) included the *met15∆0* allele, whereas the BY4742 genome (target) included *MET15*. In these samples, the GD clones contained both alleles, also supporting the view that these were diploid yeast.

### GD action utilizes HDR over NHEJ

**Fig. 2.**
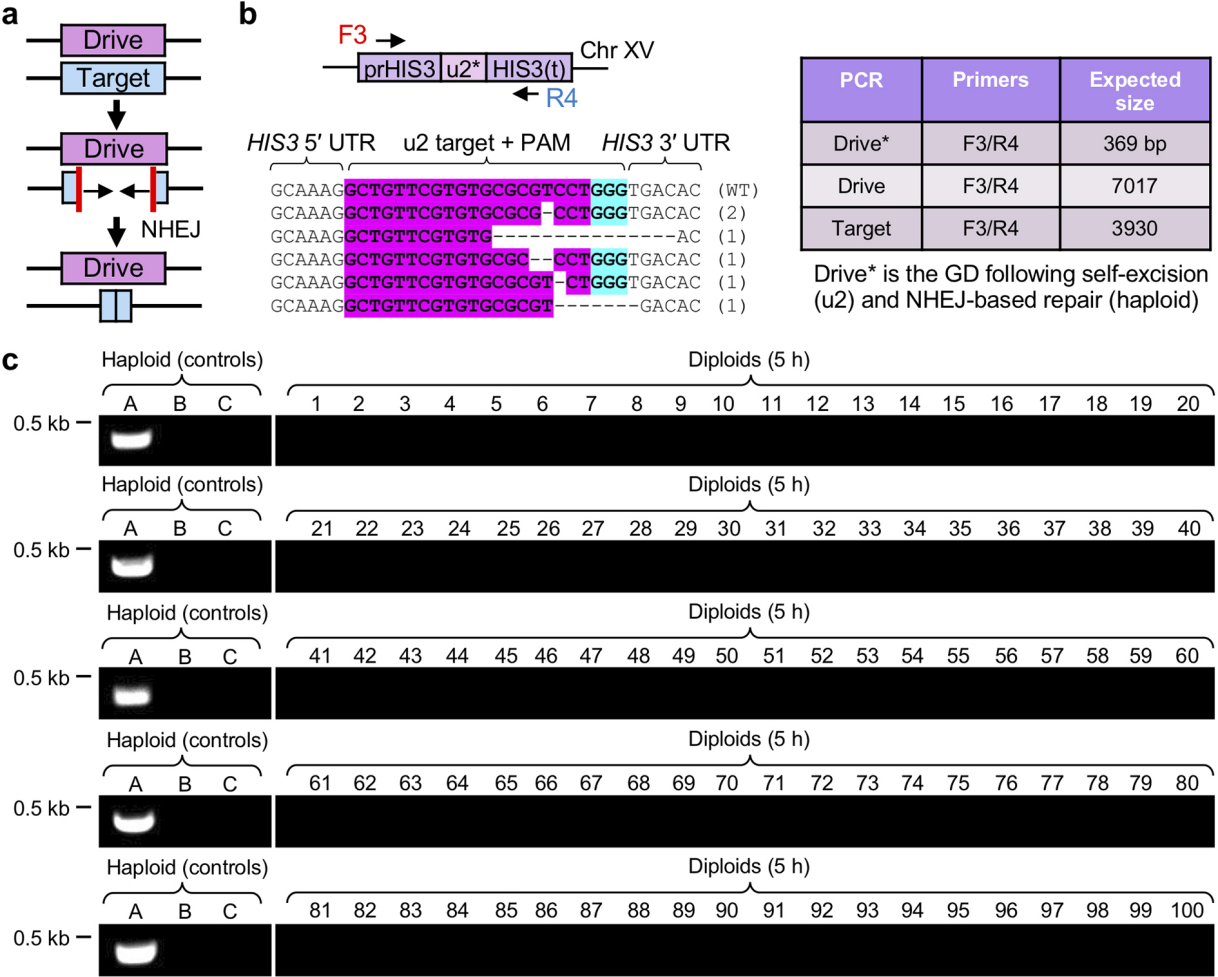
Homology-directed repair is the primary mechanism for GD action compared to NHEJ. (a) Schematic of the potential for NHEJ-based repair of the target allele that would prevent HDR-based repair and successful propagation of a GD. (b) Generation of a control allele in a haploid strain using self-cleavage of Cas9 and artificial u2 sites. The GD haploid strain from Fig. 1 (GFY-2383) was activated and a plasmid expressing sgRNA(u2) (pGF-V809) was transformed with no additional donor DNA. Surviving clonal isolates were obtained on SD-LEU medium and the *HIS3* locus was analysed. The sample sequences obtained are illustrated for six independent isolates. The expected PCR product size for NHEJ-based repair within this target allele (designed control A) would equal 369 bp (for an isolate lacking a single base at the cleavage site). Use of the same primers to amplify the entire drive or target allele would result in a product of 7017 and 3930 bp, respectively. (c) From 47 GD diploids tested (Fig. 1), 1 to 3 isolates (100 in total) were obtained at random that were resistant to G418, had lost the target allele (sensitivity to SD-HIS plates) and were diploid (via the mating test, see Fig. S3). For all strains, the *HIS3* locus was also examined by diagnostic PCR (Fig. S5). Vertical white lines were added for clarity. Control B and control C (haploid) strains from Fig. 1 were used.

Studies with CRISPR GD systems in insects have found that a competing DNA repair pathway, NHEJ, can provide a source of drive-resistant alleles [20, 32, 33]. This occurs due to the following mechanism of repair: the nuclease induces a DSB within the target, yet rather than the drive coping and replacing the target locus through HDR, the cleaved chromosome repairs via NHEJ, including destruction of the original CRISPR site(s) (Fig. 2a). Give that our yeast GD1 includes two identical (u1′) sites flanking the entire target locus, cleavage followed by NHEJ-based repair would result in complete excision of the target DNA. This mode of action was demonstrated within haploids harbouring GD1 at the *HIS3* locus and a guide RNA that targeted the flanking (u2) sequences [13] (Fig. 2b). In haploid cells, Cas9 was induced through culturing haploid yeast with galactose and the high-copy sgRNA(u2)-containing plasmid was transformed followed by selection and recovery on dextrose-containing plates. Surviving clones were then tested for loss of G418 resistance (indicating removal of the entire GD locus and Kan^R^ selection marker) and analysed using PCR and DNA sequencing. Independent clones had indels at the site of cleavage +3 bp upstream of the 5′ end of the PAM within the remaining (u2) CRISPR site (Fig. 2b). These surviving haploid strains had excised the GD locus; one such isolate served as a control for analysis of possible NHEJ-based repair within our GD system.

From our first-generation CRISPR drive experiments, we randomly selected 100 independent clones across 3 trials involving 47 separately generated diploid strains between the drive and target parental strains (Fig. 1). These were tested as clonal diploids (using the mating test) that were also G418-resistant and sensitive to the SD-HIS condition; diagnostic PCRs were also performed, demonstrating that these isolates had maintained the drive and lost the target allele (Fig. S5). We next performed a PCR using oligonucleotides to the *HIS3* promoter and terminator for all 100 strains compared to controls (Fig. 2c). For the haploid strain containing a self-cleaved *HIS3* locus and a modified (u2) site from NHEJ-based repair (Fig. 2b), a small PCR fragment was generated (369 bp). PCR conditions were optimized for the generation of this fragment size. However, for all 100 separate drives that had successfully lost the target allele, none produced a band of similar size, indicating that the locus had been replaced by the drive, rather than repaired by NHEJ. A similar near-100% effectiveness of a yeast GD was also observed in a previous CRISPR system using sporulation of diploids and subsequent testing of generated haploids [6]. While it remains possible that NHEJ may still occur within this yeast-based system, it may be significantly below that of HDR, given the effectiveness of homologous recombination in budding yeast.

### Multiplexing within the target locus

**Fig. 3.**
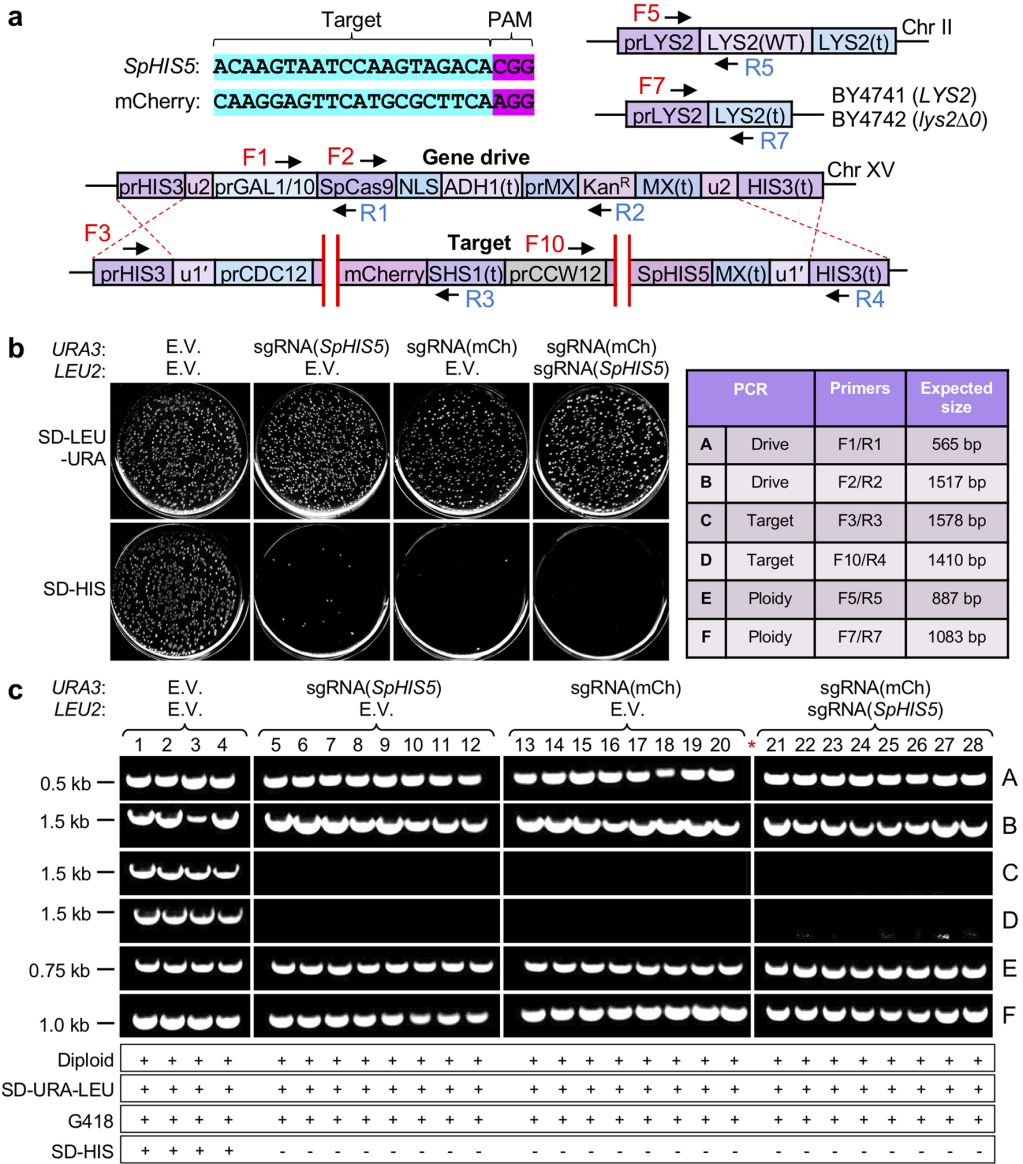
Use of alternative sgRNAs within the target allele allows for successful CRISPR GD action. (a) Schematic of a modified drive/target system that included target sites within the mCherry and *SpHIS5* coding sequences. Cross-over between the MX(t) DNA is also possible upstream of the (u1’) and (u2) sequences, but is not illustrated for clarity. (b) Two high-copy plasmids (*URA3*-based, pGF-V2153, pGF-V2159; *LEU2*-based, pGF-V2152) were included within the drive strain GFY-2383. EV, empty pRS425 or pRS426 vectors. Following activation in galactose, diploids were plated as in Fig. 1 using SD-LEU-URA and SD-HIS medium. Individual plate images (full plates) were edited for contrast. (c) Diagnostic PCRs of clonal diploid isolates from (c). Top right: table of primers chosen for analysis and the expected product sizes. Horizontal white lines designate separate DNA gels. Vertical white lines are included for clarity; red asterisk, a separate DNA gel was used for samples 21–28.

Our GD1 design utilized flanking (u2) or (u1′) sites surrounding the engineered drive or target alleles, respectively. While this increases biosafety and containment, it also allows dual cleavage using only a single guide RNA (both sites are identical sequences). Previous work has demonstrated that multiplexing to identical sites across the genome can be accomplished for both native and artificial sequences for genomic editing or recruitment of enzymatically dead Cas9 fusions [25, 34]. Moreover, multiplexing the Cas9 nuclease to the intended target has become an important strategy in insect systems to promote drive success and reduce drive resistance [20]. However, our (u1′/u2) site arrangements present a unique scenario for NHEJ-based repair. Cleavage of both sites followed by precise repair (of the 5′ end of the upstream site and the 3′ end of the downstream site) would result in recreation of a new (u1′) site that could theoretically be subjected to additional rounds of cleavage and repair. In order to test whether our GD system would still be effective when the flanking (u1′) sites were not used, we designed new guide RNAs to positions within the coding sequences of mCherry and *S. pombe HIS5* (both non-native yeast genes). We chose sequences (Fig. 3a) that still provided a maximum mismatch from the budding yeast genome for biosafety reasons and to prevent (or minimize) off-target effects.

First-generation drive strains were transformed with empty vector controls, a high-copy plasmid expressing sgRNA(mcherry), a high-copy plasmid expressing sgRNA(*SpHIS5*), or both guide RNAs; these were tested against the target strain for drive efficiency (similar to Fig. 1). The inclusion of only one guide RNA still allowed for strong GD activity, with nearly all colonies sensitive to the SD-HIS condition (Fig. 3b). We noticed that for drives that multiplexed to both target sites, the number of surviving colonies was either 0 or very close to 0 (near 100 % drive activity), a slight improvement over the same drives that utilized only a single guide; this had not been previously tested given our dual (u1)-based system. Experiments were repeated after switching the plasmid backbone (pRS426 and pRS425) for the sgRNA(mCherry) and sgRNA(*SpHIS5*) constructs and similar results were obtained (data not shown).

Subsequent analyses of selected diploid clones from each condition demonstrated the presence of the drive allele, loss of the target (for active conditions) and diploid status based on the *LYS2* locus (Fig. 3c). Of note, because the Cas9 cleavage sites in this experiment were designed to internal sequences within the target allele, it remained possible that recombination may have occurred between the drive and target MX(t) DNA segment (rather than within the flanking *HIS3* terminator). However, in both cases, the entire target locus would still be replaced by the drive allele for an active GD; the only difference would be preservation of the downstream (u1′) site (29 bp) rather than replacement by the (u2) sequence (23 bp). Diagnostic PCRs to the target locus did not discriminate between these two possibilities because the *CCW12* promoter sequence was excised in both cases (Fig. 3c, PCR-D).

For drives harbouring only one guide RNA to *SpHIS5* (isolates 5–12), it was possible that NHEJ-based repair may have allowed the formation of indels that could have disrupted the reading frame and/or introduced a premature stop codon (also providing sensitivity on SD-HIS plates and phenocopying loss of the entire allele through HDR). However, NHEJ-based repair should have still allowed for one or two amplified PCRs (Fig. 3c, PCRs C and/or D) within the target locus, given that positioning of the DNA primers was sufficiently distant from the cleavage site within *SpHIS5*. Our sampled isolates did not allow for the amplification of either PCR, demonstrating that HDR had converted the entire target locus to GD1/GD1. Finally, for isolates harbouring both guide RNA constructs (21–28), it remained possible that the intervening sequence between the cleavage sites in mCherry and *SpHIS5* (Fig. 3a) were excised and the chromosome was repaired by NHEJ (and eliminated *SpHIS5* expression and/or function). Our amplified fragments of the target locus (PCRs C and D) both relied on primers for sequences that may have been excised in this scenario. Therefore, we also tested for amplification of the target locus (isolates 21–28) for the presence of pr*CDC12* positioned at the *HIS3* locus; we could not detect any evidence supporting NHEJ-based repair for these sampled isolates (Fig. S6).

**Fig. 4.**
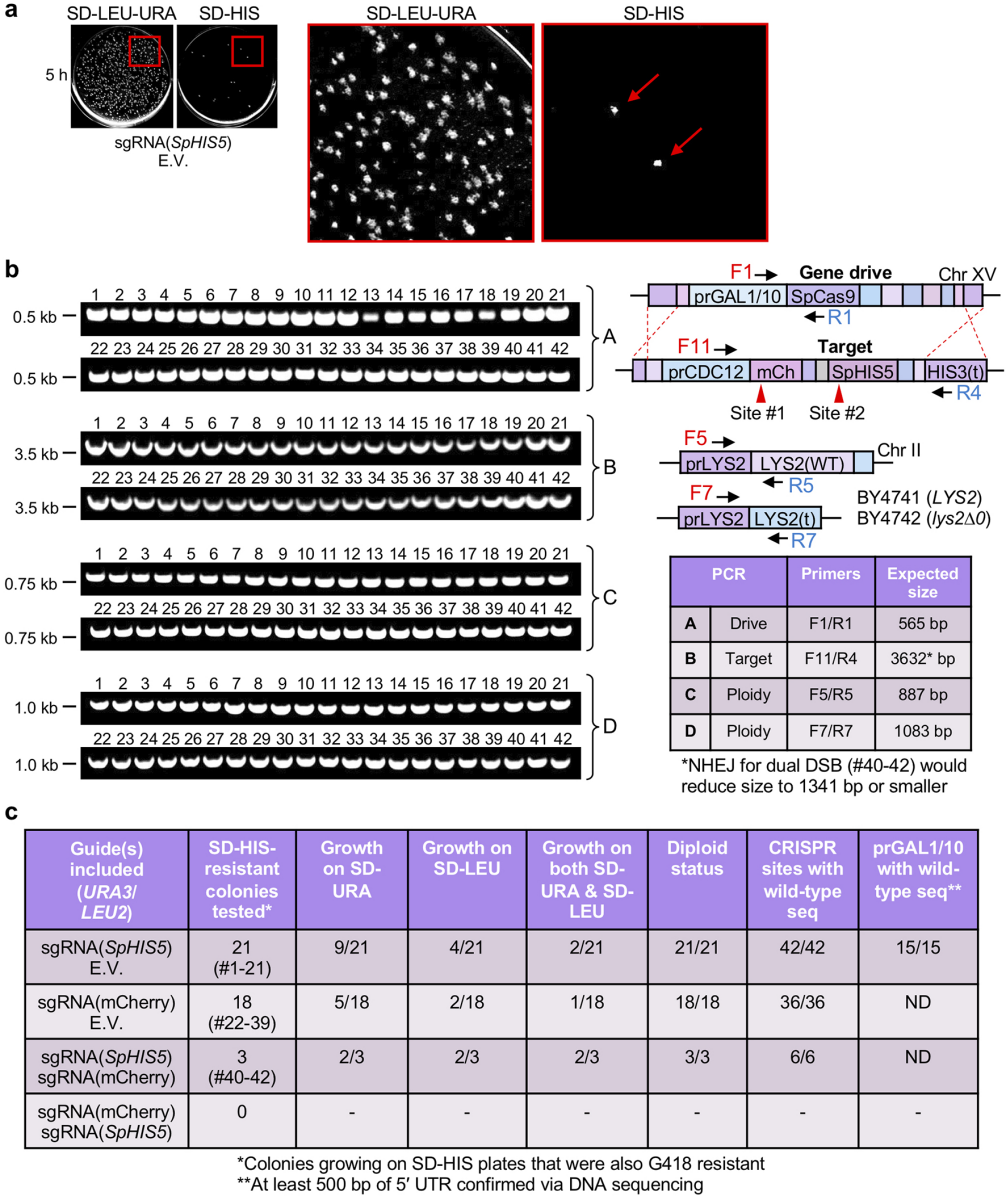
Analysis of yeast isolates still displaying growth on SD-HIS medium following GD activation. (a) Example plates from Fig. 3 are illustrated with an area highlighted, enlarged for clarity (right) and edited for increased contrast (each plate separately). Red arrows illustrate colonies resistant to the SD-HIS condition. (b) Forty-two separate clonal isolates were obtained from independent SD-HIS plates used for GDs in Fig. 3 that were also resistant to G418. Diagnostic PCRs were performed on purified chromosomal DNA for both the *HIS3* and *LYS2* loci. Asterisk, for isolates 40–42, if NHEJ had repaired the *HIS3* locus following dual cleavage, the amplified product size (PCR-B) would be approximately 1341 bp (depending on included indels). (c) Yeast clones still displaying growth on SD-HIS from GD experiments from Fig. 3 (that were also resistant to G418) were subjected to additional growth and ploidy tests, diagnostic PCRs and DNA sequencing. nd, not determined. Sequencing of the mCherry and *SpHIS5* target sites included confirmation of several hundred bases upstream and downstream of the site at minimum. Fifteen of the 21 isolates from the sgRNA(*SpHIS5*) condition were chosen for sequencing of the *GAL1/10* promoter.

From all our work involving CRISPR drives in yeast [12, 13, 17, 23, 24], we have observed that there are typically a very small number of surviving colonies on the SD-HIS condition, regardless of the choice of guide RNA (Figs 1 and 3). It is important to note that our developed GD system does not impose selection or challenge concurrent with drive action. There is no (or little) selective advantage or disadvantage to maintaining or losing the target locus (cells were allowed to recover on SD-LEU or SD-URA-LEU plates to select for any included plasmids prior to examination on SD-HIS). This system is distinct from other types of GDs that might directly select for loss of the target allele (for example, loss of *URA3* for resistance to 5-fluoroorotic acid) or include selective pressure within the population directly dependent on drive action and the subsequent outcome of the progeny (for example, sex determination bias or alteration of traits required for optimal fitness). Our current system does not provide additional challenge to ‘resist’ at the point of drive action and repair. Therefore, we expected that our drives would allow for near 100 % activity under standard conditions. However, to better understand the occurrence of this small percentage of ‘resistant’ clones that still maintained growth on SD-HIS plates, we analysed 42 independent diploid isolates from the drives that utilized mCherry and *S. pombe HIS5* targets and were still G418-resistant (drive allele) and SD-HIS-resistant (target allele) (Fig. 4). Following growth on SD-HIS plates (Fig. 4a), yeast were selected as clonal isolates (a second round of selection on SD-HIS) and chromosomal DNA was isolated and subjected to diagnostic PCRs of the *HIS3* and *LYS2* loci; all 42 strains were diploids and still contained both the drive and target cassettes (Fig. 4b). The target loci were amplified and sequenced surrounding both the mCherry and *S. pombe HIS5* sites for all clones. Interestingly, we confirmed that all 42 strains had unmodified sequences at both CRISPR sites, including several hundred bases upstream and downstream of the intended cleavage site identical to the original haploid parental strain. Moreover, for 15 isolates from the sgRNA(*SpHIS5*) condition, the *GAL1/10* promoter within the drive allele was also sequenced and did not contain any alterations (Fig. 4c). Of note, for the three resistant isolates that originally included both guide RNAs, the intervening sequence between target sites was maintained (Fig. 4c 40–42). Finally, we tested for the occurrence of the *URA3*-based and *LEU2*-based high-copy plasmids harbouring guide RNA cassettes. For some isolates, one or both plasmids were lost in the absence of continual selection (Fig. 4c). However, loss of the guide RNA plasmids has been previously demonstrated in our system and other yeast GDs [6, 13]. From these data, we conclude that it is unlikely that NHEJ (through formation of indels) or *de novo* mutation of the target site(s) could account for the accumulation of some resistant yeast colonies following the action of our GD.

### A second-generation CRISPR drive and imperfect nuclease activation

**Fig. 5.**
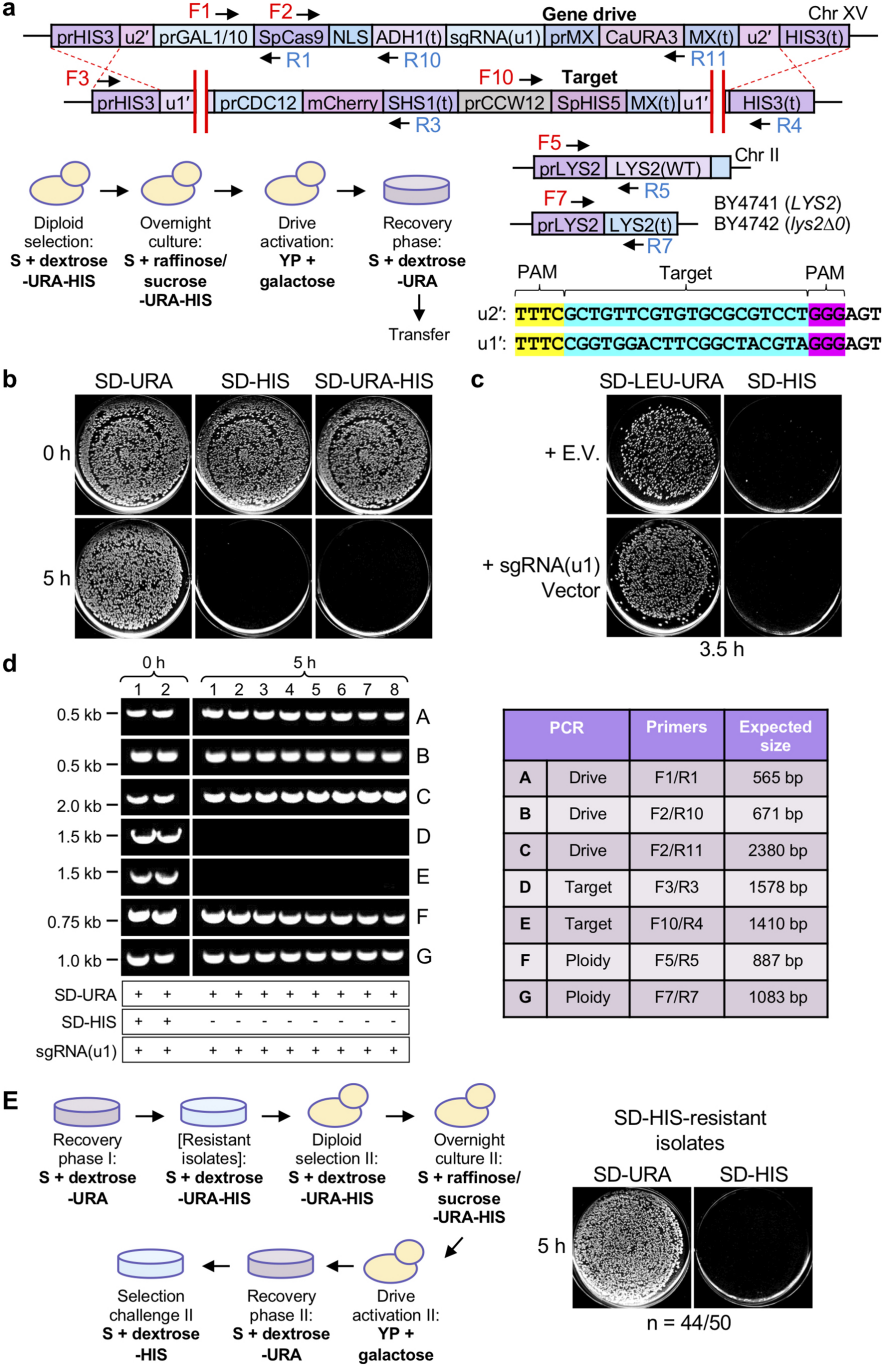
A second-generation CRISPR GD includes an integrated sgRNA cassette. (a) An updated GD system includes a number of alterations: (i) an integrated sgRNA(u1) expression cassette downstream of the *ADH1* terminator, (ii) *C. albicans URA3* as a selection marker for the drive locus (as opposed to Kan^R^) and (iii) a modified (u2’) site similar to u1’ (includes a 5′-TTTV-3′ sequence at the 5’ end that is compatible with Cas12a systems). Changes to the activation protocol include dual selection for both drive/target loci (SD-URA-HIS) during diploid selection as well as preinduction medium. The recovery phase maintains selection for the drive allele on SD-URA plates. (b) Strains GFY-4325 and 4326 were mated with GFY-3733, diploids were selected and Cas9 expression was activated by culturing for 5 h in galactose. Following recovery on dextrose, colonies were transferred to both SD-HIS and SD-URA-HIS plates. (c) Drive strains were transformed with either an empty pRS425 vector or a sgRNA(u1)-expressing cassette (pGF-V1220), diploids were selected on SD-LEU-URA-HIS and GDs were activated for 3.5 h in galactose before plating. (d) Left: clonal isolates following drive activation (b) were tested by diagnostic PCRs. Right: table of examined primer combinations and the expected amplified product sizes. The presence of the integrated sgRNA(u1) cassette was determined by PCR and DNA sequencing for all 10 isolates. Horizontal white lines indicate separate DNA gels used; a vertical white line was included for clarity. (e) Across 12 independent GD2 diploid strains activated (b), a total of 50 clonal isolates that maintained growth on SD-URA-HIS were collected. Left: strains were grown on SD-URA-HIS plates a second time and prepared for a second round of GD activation. Right: representative plates of one isolate that was originally resistant to the SD-URA-HIS condition (44/50). All agar plates were edited for contrast and clarity (each plate separately).

The design of eukaryotic drives includes placement of both the nuclease and corresponding guide RNA expression cassette(s) at the intended locus (or loci) of interest. In our yeast system, we could rely on plasmid-borne expression for guide RNAs. While this is convenient for the testing of multiple guide sequences, altered variants, multiplexing and biosafety (rapid loss without selection), it does not examine the action of a GD harbouring both critical components within the genome. Therefore, we developed a second-generation CRISPR drive (GD2) in yeast to test the effectiveness of this altered arrangement. The modifications to our original drive design (Fig. 1) included the following: (i) new (u2′) sites flanking the GD that included additional PAM sequences for *F. novicida* Cas12a and *S. aureus* Cas9; (ii) an integrated guide RNA cassette downstream of the *S. pyogenes* Cas9 terminator sequence; and (iii) switching of the Kan^R^ selection marker for the *Candida albicans URA3* marker. Moreover, we adjusted our methodology for selection and activation of the GD to include continual selection for the drive and target alleles (using media lacking both uracil and histidine) within our preinduction culture and continual selection for the drive allele within the recovery plate following galactose treatment (Fig. 5a). Using this system, we tested yeast colonies on both SD-URA-HIS and SD-HIS plates following a 5 h galactose induction of Cas9. We found no difference between these two conditions; both resulted in nearly 100 % active drives (Fig. 5b). We also tested whether integration of the guide RNA expression cassette might result in a less efficient drive system (due to limiting sgRNA expression). We included either an empty vector control or second copy of the sgRNA(u1)-expressing high-copy plasmid within GD2 and utilized a 3.5 h galactose induction. We observed only a minimal difference between one integrated guide cassette and a second cassette on a high-copy plasmid, although this may be specific to the *SNR52* promoter, the *S. pyogenes* Cas9 nuclease and/or use of the *HIS3* locus for our drive system (Fig. 5c). Analysis of clonal isolates following drive activation demonstrated the presence of the drive allele (PCRs A-C), loss of the target allele (PCRs D,E), diploid status using *LYS2* (PCRs F,G) and also the presence of the guide RNA cassette itself (DNA sequencing) proximal to the Cas9 gene (Fig. 5d).

Finally, given that the guide RNA expression cassette was integrated within the drive, we examined 50 separate resistant isolates across multiple plates that maintained survival on the SD-URA-HIS condition. It is important to note that these sampled 50 clones represent only a very small percentage of all colonies examined following drive activation (Fig. 5b). Yeast were selected as clonal isolates on the same media type once more – selecting for one marker within the drive (*C. albicans URA3*) and one marker within the target (*S. pombe HIS5*). Next, strains were preinduced overnight, cultured in galactose a second time for 5 h, recovered on SD-URA plates and tested once more on SD-HIS (Fig. 5e). This experiment examined whether the remaining resistant clones were still competent to express Cas9 and function as an active drive. Interestingly, 88 % of the 50 clones (44/50) were able to reactivate the drive to near 100 % activity, demonstrating that imperfect expression (or function) of the nuclease could explain the occurrence of at least some remaining heterozygous clones (Fig. 5e).

## Discussion

### A model system for study of GD action

Our yeast GD model includes a number of important benefits: simplicity, genetic tractability, tunability and biosafety. While previous work with our initial GD1 system highlighted a number of variations to guide RNA sequences [13], Cas9 subcellular localization [13] and anti-CRISPR-based inhibition [12], numerous other alterations to drive action (Cas9 enzymatic activity), drive control/regulation (anti-CRISPRs or nuclease degradation) and DNA repair could be tested in future iterations. Our findings from this study included controls to allow for efficient examination of diploid status (amplification of the *LYS2* and/or *MET15* loci), comparisons to parental haploid genomes and the lack of detectable NHEJ-based repair within our GD, regardless of the choice of target sites. We recognize that NHEJ-based repair may still occur within our yeast model, but this will require the development of a distinct drive/target system for simultaneous detection of (i) loss of the target allele and (ii) maintenance of only a *single* copy of the drive allele within a diploid genome. HDR and/or NHEJ repair systems themselves could also be examined within our drive system; this might allow for future modulation of DNA repair to aid in drive optimization or control [24].

### Optimizing nuclease expression *in vivo*

Our study also examined a large number of isolated resistant colonies that continued to be present within our yeast system on the final medium (SD-HIS plates), albeit at a small percentage within sampled populations of asynchronous cultures. Various sources can provide alterations within the target DNA that will prevent action of the drive (and develop true resistance), including *de novo* mutation [32], sequence variation within a mixed population [35] and NHEJ-based repair followed by indel formation or DNA excision [20]. In these cases, the nuclease would be unable to successfully cleave the altered target site(s) in subsequent generations and this would prevent *super*-Mendelian inheritance. In our model, we examined the target loci of 42 clonal diploid isolates still harbouring both the drive and target alleles following drive activation; we did not detect any incidence of mutation within the 23 bp target site and PAM as well as a large amount of flanking DNA. We suspected that one explanation for the formation of these resistant colonies was failed or poor activation of Cas9 (rather than inappropriate mutation or repair of the target site). Our data suggest that this is the case for many *SpHIS5*-positive clones as a second round of galactose culturing provided successful GD action in 88 % of isolates from our GD2 system. Further, the presence of the MX terminator (Fig. 5) within both the drive and target locus upstream of the (u1′) cut site may provide a source of inappropriate crossover if there is also lack of cleavage on the upstream (u1′) position. The goal of obtaining a 100 % effective GD may be useful in some scenarios; however, even GDs that allow for resistance may still be effective at spreading within natural populations [36]. Future iterations of our drive/target system may rely on targeting of the mCherry and *SpHIS5* coding sequences (or others), given the success of multiplexing to these sites. It may also be possible to further optimize our yeast drives (and activation of the nuclease) by including a selective advantage or requirement for Cas9 expression coupled to survival of an environmental challenge through translational fusions or *GAL1/10-*dependent transcription of other markers.

Our GD model provides a highly tractable system to explore alternative drive arrangements [split drives [14, 24], daisy chain drives [37], nuclease inhibitors (small molecules [15] or naturally evolved anti-CRISPRs [12]) and DNA repair systems [24]]. Future work may aid in informing complex drive designs, arrangements to reduce or counter resistance, or safe practices for study or possible application in higher eukaryotes.

## Supplementary Data

Supplementary material 1
