# Supplementary material for: Analysis of CRISPR gene drive design in budding yeast
Source: Access Microbiol. 2019 Sep 11;1(9):e000059. doi: 10.1099/acmi.0.000059 (PMC7472540; doi:10.1099/acmi.0.000059)
Supplement: Supplementary material 1 [file acmi-1-059-s001.pdf]

SUPPLEMENTARY INFORMATION

For

Analysis of CRISPR gene drive design in budding yeast

Yao Yan[1] and Gregory C. Finnigan[1*]

[1]Department of Biochemistry and Molecular Biophysics, Kansas State University, 141 Chalmers Hall, Manhattan, KS 66506 USA

*Correspondence to: Gregory C. Finnigan, Phone: (785) 532-6939; FAX; (785) 532-7278;

E-mail: gfinnigan@ksu.edu

Table S1. Yeast strains used in this study.

Strain	Genotype	Reference
BY4741	*MAT***a** *his3Δ1 leu2Δ0 met15Δ0 ura3Δ0 LYS2*	1
BY4742	*MATα his3Δ1 leu2Δ0 lys2Δ0 ura3Δ0 MET15*	1
GFY-150[a]	BY4742; *cdc11Δ::prMX::Kan^R::MX(t)* + pJT1520 (*prCDC11::CDC11*)	This study
GFY-153[a]	BY4741; *cdc11Δ::prMX::Kan^R::MX(t)* + pJT1520 (*prCDC11::CDC11*)	This study
GFY-2383	BY4741; *his3Δ::prHIS3:(u2)::prGAL1/10::SpCas9::NLS::ADH1(t):prMX::Kan^R::MX(t)::(u2)::HIS3(t)*	2
GFY-3733[b]	BY4742; *his3Δ::prHIS3::(u1')::prCDC12::mCherry::SHS1(t)::prCCW12::SpHIS5::MX(t)::(u1')::HIS3(t)*	This study
GFY-4325, GFY-4326[c]	BY4741; *his3Δ::prHIS3:(u2')::prGAL1/10::SpCas9::NLS::ADH1(t)::sgRNA(u1)::prMX::CaURA3::MX(t)::(u2')::HIS3(t)*	This study

[a]Strains GFY-150 and GFY-153 were generated from a previous study[3]. GFY-153 was the parental strain to generate GFY-163. Briefly, the *CDC11* locus was replaced by the Kan^R deletion cassette in BY4741 or BY4742 WT yeast harboring a *URA3*-based covering vector (pJT1520[4]) that expresses a copy of *CDC11* under its native promoter. These strains were used in mating tests (Fig. S3).

[b]Strain GFY-3733 is similar to GFY-3207. Artificial CRISPR sites[5] (u1') are positioned flanking the entire cassette with the sequence 5'-T**TTTC**CGGTGGACTTCGGCTACGTA**GGG**AGT-3'. The bold and underlined sequences include the PAM sites for Cas12a/Cpf1 (TTTV at the 5' end) and *S. pyogenes* Cas9 (NGG at the 3' end) with a common target site. Strain GFY-3733 was generated from integration of the cassette from the pGF-IVL1511 vector at the *HIS3* locus in BY4742 yeast. The *HIS5* gene is from fission yeast *S. pombe* and is the functional equivalent of *S. cerevisiae HIS3*. The 5' T for the upstream (u1') site has been artificially added. For the downstream (u1') site, the T already existed within the MX(t) sequence. For the 3' T within the upstream (u1') site, this base was artificially added. For the downstream (u1') site, this T already existed as part of the *HIS3*(t) sequence.

[c]Strains GFY-4325 and GFY-4326 were two separate isolates created in an identical manner. First, BY4741 yeast were transformed with six overlapping PCR fragments that were assembled *in vivo* through selection on rich medium containing G418 (the initial integration contained the prMX-Kan^R-MX(t) drug resistance cassette). Second, CRISPR-based editing was performed by activating Cas9 expression (galactose metabolism) and co-transformation of the pGF-V1642 plasmid expressing the sgRNA(Kan^R) cassette with a PCR fragment, prMX-*CaURA3*-MX(t) (amplified from plasmid JT2869), to serve as donor DNA. The *URA3* gene is from *C. albicans*. Colonies were selected that grew on SD-LEU. Clonal isolates were tested for loss of G418 resistance and also survival on SD-URA plates. Third, yeast were maintained on non-selection media (multiple plates) to allow for loss of the high-copy sgRNA(Kan^R) plasmid (and sensitivity on SD-LEU plates). The final strains were confirmed by diagnostic PCR and Sanger DNA sequencing. This strain includes a 431 bp expression cassette for the *S. pyogenes* sgRNA to target (u1') immediately following the *ADH1*(t) sequence. Additionally, a modified (u2') site was included (similar to the (u2) sites present within strain GFY-2383) with the artificial sequence 5'-T**TTTC**GCTGTTCGTGTGCGCGTCCT**GGG**AGT-3'. Dual sequences for *F. novicida* Cas12a/Cpf1 and *S. pyogenes* Cas9 (PAMs) are in bold and underlined text, respectively.

Table S2. Plasmids used in this study.

Plasmid	Description	Reference
pRS425	2μ, *LEU2*	6
pRS426	2μ, *URA3*	6
pRS313	CEN, *HIS3*	7
pRS315	CEN, *LEU2*	7
pGF-V1220[a]	pRS425; *prSNR52::Sp-sgRNA(u1)::SUP4(t)*	2
pGF-V809[b]	pRS425; *prSNR52::Sp-sgRNA(u2)::SUP4(t)*	2
pGF-V1642[c]	pRS425; *prSNR52::Sp-sgRNA(Kan^R)::SUP4(t)*	8
pGF-V2152[d]	pRS425; *prSNR52::Sp-sgRNA(SpHIS5)::SUP4(t)*	This study
pGF-V2153	pRS426; *prSNR52::Sp-sgRNA(SpHIS5)::SUP4(t)*	This study
pGF-V2158[e]	pRS425; *prSNR52::Sp-sgRNA(mCherry)::SUP4(t)*	This study
pGF-V2159	pRS426; *prSNR52::Sp-sgRNA(mCherry)::SUP4(t)*	This study

[a]The (u1/u1') target sequence is 5'-CGGTGGACTTCGGCTACGTA-3'. The guide RNA constructs were modeled from a previous study[9] and contain 269 bp of the *SNR52* promoter, a 79

bp tracrRNA, and the *SUP4* terminator (20 bp) along with variable flanking sequence (approximately 25-35 bp). The sgRNA(u1) is able to target both (u1) and (u1') sequences.

[b]The sgRNA(u2) target sequence is 5'-GCTGTTCGTGTGCGCGTCCT-3'. The sgRNA(u2) is able to target both (u2) and (u2') sequences.

[c]The sgRNA(Kan^R) target sequence is 5'-GCCATCCTATGGAACTGCCT-3'. An alternative name to this vector is "pGF-425+1275."

[d]The sgRNA(*SpHIS5*) target sequence is 5'-ACAAGTAATCCAAGTAGACA-3'. The entire cassette was synthesized (GenScript) as a custom gene into a pUC57 (Kan^R) vector and sub-cloned to pRS425/pRS426 using unique flanking restriction sites.

[e]The sgRNA(mCherry) target sequence is 5'-CAAGGAGTTCATGCGCTTCA-3'. The expression cassette was generated similar to pGF-V1642 using *in vivo* plasmid assembly[10] and unique overlapping oligonucleotides to generate the unique 20 bp guide sequence.[8] Second, the entire cassette was PCR amplified (from pGF-IVL1277) and cloned into the pCR™Blunt II-TOPO® vector (Life Technologies). Third, the cassette was sub-cloned to pRS425/pRS426 using unique restriction sites.

Table S3. Oligonucleotides used in this study.

**Oligonucleotide Name**	**DNA Sequence (5' to 3')**
F1: Int pr*GAL1/10* +192 F	GGGGTAATTAATCAGCGAAGCGATGATTTTTG
R1: Int *S.p.Cas9* +373 R	CATCAACGATGTTACCGAAGATTGGATGTC
F2: Int *S.p.Cas9* +3653 F	CGGTAGAAAAAGAATGTTAGCTTCAGCTGG
R2: Int Kan R	GAACACTGCCAGCGCATCAACAATATTTTC
F3: pr*HIS3* +196 F	GGCCTCCTCTAGTACACTCTATATTTTTTTATGC
R3: *SHS1*(t) -192 R	GCCATATTTAAATTTATCCCTACAATTATTTGACACTGTTTG
F4: Int *SpHIS5* F1	GGGAGAACAAGTAATCCAAGTAGACACGGG
R4: *HIS3*(t) -151 R	CGCCTCGTTCAGAATGACACGTATAGAATG
F5: pr*LYS2* +258 F	CAATAGTTTTGCCAGCGGAATTCCACTTGC
R5: *LYS2* Int +629 R	GTTATGCAATTGGATGGATCGCTTAGCGC
F6: *LYS2* Int +3796 F	GACTACTTGTATACTTTACACGATTATGGTTACGATG
R6: *LYS2*(t) -367 R	TTCATTTTAGACCCATGGTGGAACCCTAGTG
F7: pr*LYS2* +636 F	GGTAAGTATGCTCATCAATCGTTCGGACTC

R7: *LYS2*(t) -755 R	CGGGCTAAGTATCGATTTGTCTCAACCTGC
F8: *MET15* Int +1253 F	TCTGGTGTTACCAAGGACTTAATTCGTGTCTC
R8: *MET15*(t) -469 R	CGATGGAATTCCAACAGCTTTACTAATCTTTACTTG
F9: pr*MET15* +1066 F	CCACAAAGCTACGAAAATTTGAAGAAAGGTTCC
R9: *MET15*(t) -897 R	CATCTTATAGGACATATTAAACTATGACGACATTGTTGC
F10: Int pr*CCW12* F	CGTACAAGTATTTCTCAGGAGTAAAAAAACCGTTTG
R10: Int *ADH1*(t) R new	CCTGACCTACAGGAAAGAGTTACTCAAGAATAAG
F11: pr*CDC12* +377 F	TGACATTCTGCAAGCTCTTTGAATCTTCCTCAAAA
R11: Ca*URA3* clone out R	TTATAATTGGCCAGTCTTTTTCAAATAAGCATTCCAAC
F12: pr*CDC12* +276 F	GATGGGACATGATGCAGTATCACGATTAGCAA

Figure S1. Yeast strains used in this study.

***LYS2* (from BY4741 background)**
prLYS2::***LYS2(WT)***::*LYS2(t)*

TACGGTGGTACCTTTTTGAACTTCGTCTCTTATGCTGCAGGAGCCGTTAGGTTAGCCGCCTTGTCTGGTAATCCAGT
CATTTGGGTTGCAACACATGACTCTATCGGGCTTGGTGAGGATGGTCCAACGCACCAACCTATTGAAACTCTGGCTC
ACTTGAGGGCTATTCCAAACATGCATGTATGGAGACCTGCTGATGGTAACGAAACTTCTGCTGCGTATTATTCTGCT
ATCAAATCTGGTCGAACACCATCTGTTGTGGCTTTATCACGACAGAATCTTCCTCAATTGGAGCATTCCTCTTTTGA
AAAAGCCTTGAAGGGTGGCTATGTGATCCATGACGTGGAGAATCCTGATATTATCCTGGTGTCAACAGGATCAGAAG
TCTCCATTTCTATAGATGCAGCCAAAAAATTGTACGATACTAAAAAAATCAAAGCAAGAGTTGTTTCCCTGCCAGAC
TTTTATACTTTTGACAGGCAAAGTGAAGAATACAGATTCTCTGTTCTACCAGACGGTGTTCCGATCATGTCCTTTGA
AGTATTGGCTACTTCAAGCTGGGGTAAGTATGCTCATCAATCGTTCGGACTCGACGAATTTGGTCGTTCAGGCAAGG
GGCCTGAAATTTACAAATTGTTCGATTTCACAGCGGACGGTGTTGCGTCAAGGGCTGAAAAGACAATCAATTACTAC
AAAGGAAAGCAGTTGCTTTCTCCTATGGGAAGAGCTTTCTAAGTCTGAAGAAGTAAACAGTTCTTTGCTATTTCACA
CTTCCTGGTTGATGGTCACTTGCTGCCTGAAATATATATATATGTATGACATATGTACTTGTTTTCTTTTTTGTGCC
TTTGTTACGTCTATATTCATTGAAACTGATTATTCGATTTTCTTCTTGCTGACCGCTTCTAGAGGCATCGCACAGTT
TTAGCGAGGAAAACTCTTCAATAGTTTTGCCAGCGGAATTCCACTTGCAATTACATAAAAAATTCCGGCGGTTTTTC
GCGTGTGACTCAATGTCGAAATACCTGCCTAATGAACATGAACATCGCCCAAATGTATTTGAAGACCCGCTGGGAGA
AGTTCAAGATATATAAGTAACAAGCAGCCAATAGTATAAAAAAAAATCTGAGTTTATTACCTTTCCTGGAATTTCAG
TGAAAAACTGCTAATTATAGAGAGATATCACAGAGTTACTCACTA**ATGACTAACGAAAAGGTCTGGATAGAGAAGTT**
GGATAATCCAACTCTTTCAGTGTTACCACATGACTTTTTACGCCCACAACAAGAACCTTATACGAAACAAGCTACAT
ATTCGTTACAGCTACCTCAGCTCGATGTGCCTCATGATAGTTTTTCTAACAAATACGCTGTCGCTTTGAGTGTATGG
GCTGCATTGATATATAGAGTAACCGGTGACGATGATATTGTTCTTTATATTGCGAATAACAAAATCTTAAGATTCAA
TATTCAACCAACGTGGTCATTTAATGAGCTGTATTCTACAATTAACAATGAGTTGAACAAGCTCAATTCTATTGAGG
CCAATTTTTCCTTTGACGAGCTAGCTGAAAAAATTCAAAGTTGCCAAGATCTGGAAAGGACCCCTCAGTTGTTCCGT
TTGGCCTTTTTGGAAAACCAAGATTTCAAATTAGACGAGTTCAAGCATCATTTAGTGGACTTTGCTTTGAATTTGGA
TACCAGTAATAATGCGCATGTTTTGAACTTAATTTATAACAGCTTACTGTATTCGAATGAAAGAGTAACCATTGTTG
CGGACCAATTTACTCAATATTTGACTGCTGCGCTAAGCGATCCATCCAATTGCATAACTAAAATCTCTCTGATCACC
GCATCATCCAAGGATAGTTTACCTGATCCAACTAAGAACTTGGGCTGGTGCGATTTCGTGGGGTGTATTCACGACAT
TTTCCAGGACAATGCTGAAGCCTTCCCAGAGAGAACCTGTGTTGTGGAGACTCCAACACTAAATTCCGACAAGTCCC
GTTCTTTCACTTATCGCGACATCAACCGCACTTCTAACATAGTTGCCCATTATTTGATTAAAACAGGTATCAAAAGA
GGTGATGTAGTGATGATCTATTCTTCTAGGGGTGTGGATTTGATGGTATGTGTGATGGGTGTCTTGAAAGCCGGCGC
AACCTTTTCAGTTATCGACCCTGCATATCCCCCAGCCAGACAAACCATTTACTTAGGTGTTGCTAAACCACGTGGGT
TGATTGTTATTAGAGCTGCTGGACAATTGGATCAACTAGTAGAAGATTACATCAATGATGAATTGGAGATTGTTTCA
AGAATCAATTCCATCGCTATTCAAGAAAATGGTACCATTGAAGGTGGCAAATTGGACAATGGCGAGGATGTTTTGGC
TCCATATGATCACTACAAAGACACCAGAACAGGTGTTGTAGTTGGACCAGATTCCAACCCAACCCTATCTTTCACAT
CTGGTTCCGAAGGTATTCCTAAGGGTGTTCTTGGTAGACATTTTTCCTTGGCTTATTATTTCAATTGGATGTCCAAA
AGGTTCAACTTAACAGAAAATGATAAATTCACAATGCTGAGCGGTATTGCACATGATCCAATTCAAAGAGATATGTT
TACACCATTATTTTTAGGTGCCCAATTGTATGTCCCTACTCAAGATGATATTGGTACACCGGGCCGTTTAGCGGAAT
GGATGAGTAAGTATGGTTGCACAGTTACCCATTTAACACCTGCCATGGGTCAATTACTTACTGCCCAAGCTACTACA
CCATTCCCTAAGTTACATCATGCGTTCTTTGTGGGTGACATTTTAACAAAACGTGATTGTCTGAGGTTACAAACCTT
GGCAGAAAATTGCCGTATTGTTAATATGTACGGTACCACTGAAACACAGCGTGCAGTTTCTTATTTCGAAGTTAAAT
CAAAAAATGACGATCCAAACTTTTTGAAAAAATTGAAAGATGTCATGCCTGCTGGTAAAGGTATGTTGAACGTTCAG
CTACTAGTTGTTAACAGGAACGATCGTACTCAAATATGTGGTATTGGCGAAATAGGTGAGATTTATGTTCGTGCAGG
TGGTTTGGCCGAAGGTTATAGAGGATTACCAGAATTGAATAAAGAAAAATTTGTGAACAACTGGTTTGTTGAAAAAG
ATCACTGGAATTATTTGGATAAGGATAATGGTGAACCTTGGAGACAATTCTGGTTAGGTCCAAGAGATAGATTGTAC
AGAACGGGTGATTTAGGTCGTTATCTACCAAACGGTGACTGTGAATGTTGCGGTAGGGCTGATGATCAAGTTAAAAT
TCGTGGGTTCAGAATCGAATTAGGAGAAATAGATACGCACATTTCCCAACATCCATTGGTAAGAGAAAACATTACTT
TAGTTCGCAAAAATGCCGACAATGAGCCAACATTGATCACATTTATGGTCCCAAGATTTGACAAGCCAGATGACTTG
TCTAAGTTCCAAAGTGATGTTCCAAAGGAGGTTGAAACTGACCCTATAGTTAAGGGCTTAATCGGTTACCATCTTTT
ATCCAAGGACATCAGGACTTTCTTAAAGAAAAGATTGGCTAGCTATGCTATGCCTTCCTTGATTGTGGTTATGGATA
AACTACCATTGAATCCAAATGGTAAAGTTGATAAGCCTAAACTTCAATTCCCAACTCCCAAGCAATTAAATTTGGTA
GCTGAAAATACAGTTTCTGAAACTGACGACTCTCAGTTTACCAATGTTGAGCGCGAGGTTAGAGACTTATGGTTAAG
TATATTACCTACCAAGCCAGCATCTGTATCACCAGATGATTCGTTTTTCGATTTAGGTGGTCATTCTATCTTGGCTA
CCAAAATGATTTTTACCTTAAAGAAAAAGCTGCAAGTTGATTTACCATTGGGCACAATTTTCAAGTATCCAACGATA

AAGGCCTTTGCCGCGGAAATTGACAGAATTAAATCATCGGGTGGATCATCTCAAGGTGAGGTCGTCGAAAATGTCAC
TGCAAATTATGCGGAAGACGCCAAGAAATTGGTTGAGACGCTACCAAGTTCGTACCCCTCTCGAGAATATTTTGTTG
AACCTAATAGTGCCGAAGGAAAAACAACAATTAATGTGTTTGTTACCGGTGTCACAGGATTTCTGGGCTCCTACATC
CTTGCAGATTTGTTAGGACGTTCTCCAAAGAACTACAGTTTCAAAGTGTTTGCCCACGTCAGGGCCAAGGATGAAGA
AGCTGCATTTGCAAGATTACAAAAGGCAGGTATCACCTATGGTACTTGGAACGAAAAATTTGCCTCAAATATTAAAG
TTGTATTAGGCGATTTATCTAAAAGCCAATTTGGTCTTTCAGATGAGAAGTGGATGGATTTGGCAAACACAGTTGAT
ATAATTATCCATAATGGTGCGTTAGTTCACTGGGTTTATCCATATGCCAAATTGAGGGATCCAAATGTTATTTCAAC
TATCAATGTTATGAGCTTAGCCGCCGTCGGCAAGCCAAAGTTCTTTGACTTTGTTTCCTCCACTTCTACTCTTGACA
CTGAATACTACTTTAATTTGTCAGATAAACTTGTTAGCGAAGGGAAGCCAGGCATTTTAGAATCAGACGATTTAATG
AACTCTGCAAGCGGGCTCACTGGTGGATATGGTCAGTCCAAATGGGCTGCTGAGTACATCATTAGACGTGCAGGTGA
AAGGGGCCTACGTGGGTGTATTGTCAGACCAGGTTACGTAACAGGTGCCTCTGCCAATGGTTCTTCAAACACAGATG
ATTTCTTATTGAGATTTTTGAAAGGTTCAGTCCAATTAGGTAAGATTCCAGATATCGAAAATTCCGTGAATATGGTT
CCAGTAGATCATGTTGCTCGTGTTGTTGTTGCTACGTCTTTGAATCCTCCCAAAGAAAATGAATTGGCCGTTGCTCA
AGTAACGGGTCACCCAAGAATATTATTCAAAGACTACTTGTATACTTTACACGATTATGGTTACGATGTCGAAATCG
AAAGCTATTCTAAATGGAAGAAATCATTGGAGGCGTCTGTTATTGACAGGAATGAAGAAAATGCGTTGTATCCTTTG
CTACACATGGTCTTAGACAACTTACCTGAAAGTACCAAAGCTCCGGAACTAGACGATAGGAACGCCGTGGCATCTTT
AAAGAAAGACACCGCATGGACAGGTGTTGATTGGTCTAATGGAATAGGTGTTACTCCAGAAGAGGTTGGTATATATA
TTGCATTTTTAAACAAGGTTGGATTTTTACCTCCACCAACTCATAATGACAAACTTCCACTGCCAAGTATAGAACTA
ACTCAAGCGCAAATAAGTCTAGTTGCTTCAGGTGCTGGTGCTCGTGGAAGCTCCGCAGCAGCTTAAGGTTGAGCATT
ACGTATGATATGTCCATGTACAATAATTAAATATGAATTAGGAGAAAGACTTAGCTTCTTTTCGGGTGATGTCACTT
AAAAACTCCGAGAATAATATATAATAAGAGAATAAAATATTAGTTATTGAATAAGAACTGTAAATCAGCTGGCGTTA
GTCTGCTAATGGCAGCTTCATCTTGGTTTATTGTAGCATGAATCATATTTGCCTTTTTTTCCTGTAATTCAATGATT
CTTGCTTCTATACTATCCTCAATGCAAAACCTTGTGATCTTCACAGGTCGATACTGACCAATTCTATGAACTCTATC
ACCACTTTGCCATTCAACACTAGGGTTCCACCATGGGTCTAAAATGAATACTTGCGAAGCTTCACAAAGATTCAAAG
CAACACCGCCCGCCTTTAAACTGACCAAGAAAACCTCGCATTGAATGTTGTTCATGAAATACTTGATGGTTTCATCT
CTTTGCGTCGGTGACATACTACCCTGAAGCTTCACTGTTTGAAATCCAGCTCTTTTCAATCTCCACTCTACCAGATC
CAGCATACTGGTAAACTGGGAAAACACAATGGATTTAATCGTTCTCTTGTTGCTTCTCAGTTTGTATAGTTCTTCCA
CAAGTGCTTCGATTTTCGTTGATGATTGCCACTTGCCACTCATGTTTAGACGGCTAACAATACTTTGCTTTTTGAAG
GAATCAAGGTCCACTTCCAAAGCAGGTTGAGACAAATCGATACTTAGCCCGATATGACAAACAGGACAAGTAAGTTT
ATTGTTGTTTTCCATGAAAGATTCCACATATTCTTTGATGCATAAACGACAGAACTTATGGTGACATTTAGATTCAA
TGGGCTCCTCAGCTTCATCGTTACATAATTGGCAGATCACGACGCCGATATCATCGCCAGGAAAATTGTTTAATCTT
TTCAAAACTAAATCAGGATGATCTGCCAGTTGCCTCATTCTTGTGATTAGGGTGAAAATGTTTGCATAATTGTTTAG
AACAACACCCTCCTCAACAAAGGAATTATACTTCCTTTTAGAATCTGTGTATAAACTTCTGTAAAGATCTTTTTCCT
CTTCATTGAAGAAGTCTCTCCTCACGGTAACAATTCTGGGCGGTAGACCCAAGTCATCCGCTCTTTCCACTTTAGTT
CTTCGCAGCATGATGTTTTTCAATAATGTCTGAA

***lys2*Δ0 (from BY4742 background)**
*prLYS2::**Added Sequence**::LYS2(t)*

TACGGTGGTACCTTTTTGAACTTCGTCTCTTATGCTGCAGGAGCCGTTAGGTTAGCCGCCTTGTCTGGTAATCCAGT
CATTTGGGTTGCAACACATGACTCTATCGGGCTTGGTGAGGATGGTCCAACGCACCAACCTATTGAAACTCTGGCTC
ACTTGAGGGCTATTCCAAACATGCATGTATGGAGACCTGCTGATGGTAACGAAACTTCTGCTGCGTATTATTCTGCT
ATCAAATCTGGTCGAACACCATCTGTTGTGGCTTTATCACGACAGAATCTTCCTCAATTGGAGCATTCCTCTTTTGA
AAAAGCCTTGAAGGGTGGCTATGTGATCCATGACGTGGAGAATCCTGATATTATCCTGGTGTCAACAGGATCAGAAG
TCTCCATTTCTATAGATGCAGCCAAAAAATTGTACGATACTAAAAAAATCAAAGCAAGAGTTGTTTCCCTGCCAGAC
TTTTATACTTTTGACAGGCAAAGTGAAGAATACAGATTCTCTGTTCTACCAGACGGTGTTCCGATCATGTCCTTTGA
AGTATTGGCTACTTCAAGCTGGGGTAAGTATGCTCATCAATCGTTCGGACTCGACGAATTTGGTCGTTCAGGCAAGG
GGCCTGAAATTTACAAATTGTTCGATTTCACAGCGGACGGTGTTGCGTCAAGGGCTGAAAAGACAATCAATTACTAC
AAAGGAAAGCAGTTGCTTTCTCCTATGGGAAGAGCTTTCTAAGTCTGAAGAAGTAAACAGTTCTTTGCTATTTCACA
CTTCCTGGTTGATGGTCACTTGCTGCCTGAAATATATATATATGTATGACATATGTACTTGTTTTCTTTTTTGTGCC
TTTGTTACGTCTATATTCATTGAAACTGATTATTCGATTTCTTCTTGCTGACC**TCTTCTGGATCC**TCCATGTACAAT
AATTAAATATGAATTAGGAGAAAGACTTAGCTTCTTTTCGGGTGATGTCACTTAAAAACTCCGAGAATAATATATAA
TAAGAGAATAAAATATTAGTTATTGAATAAGAACTGTAAATCAGCTGGCGTTAGTCTGCTAATGGCAGCTTCATCTT
GGTTTATTGTAGCATGAATCATATTTGCCTTTTTTTCCTGTAATTCAATGATTCTTGCTTCTATACTATCCTCAATG
CAAAACCTTGTGATCTTCACAGGTCGATACTGACCAATTCTATGAACTCTATCACCACTTTGCCATTCAACACTAGG
GTTCCACCATGGGTCTAAAATGAATACTTGCGAAGCTTCACAAAGATTCAAAGCAACACCGCCCGCCTTTAAACTGA
CCAAGAAAACCTCGCATTGAATGTTGTTCATGAAATACTTGATGGTTTCATCTCTTTGCGTCGGTGACATACTACCC

TGAAGCTTCACTGTTTGAAATCCAGCTCTTTTCAATCTCCACTCTACCAGATCCAGCATACTGGTAAACTGGGAAAA
CACAATGGATTTAATCGTTCTCTTGTTGCTTCTCAGTTTGTATAGTTCTTCCACAAGTGCTTCGATTTTCGTTGATG
ATTGCCACTTGCCACTCATGTTTAGACGGCTAACAATACTTTGCTTTTTGAAGGAATCAAGGTCCACTTCCAAAGCA
GGTTGAGACAAATCGATACTTAGCCCGATATGACAAACAGGACAAGTAAGTTTATTGTTGTTTTCCATGAAAGATTC
CACATATTCTTTGATGCATAAACGACAGAACTTATGGTGACATTTAGATTCAATGGGCTCCTCAGCTTCATCGTTAC
ATAATTGGCAGATCACGACGCCGATATCATCGCCAGGAAAATTGTTTAATCTTTTCAAAACTAAATCAGGATGATCT
GCCAGTTGCCTCATTCTTGTGATTAGGGTGAAAATGTTTGCATAATTGTTTAGAACAACACCCTCCTCAACAAAGGA
ATTATACTTCCTTTTAGAATCTGTGTATAAACTTCTGTAAAGATCTTTTTCCTCTTCATTGAAGAAGTCTCTCCTCA
CGGTAACAATTCTGGGCGGTAGACCCAAGTCATCCGCTCTTTCCACTTTAGTTCTTCGCAGCATGATGTTTTTCAAT
AATGTCTGAA

Promoter region: 299 bp of immediate 5’ UTR deleted and T at +313 upstream of start codon was deleted. “TCTTCTGGATCC” sequence added in place of *LYS2* gene. Terminator region: 23 bps of immediate 3’ UTR was deleted.

MET15 **(from BY4742 background)**
prMET15::***MET15(WT)***::*MET15(t)*

CCTTTTTACCTCATTGCACTAATAAAAAAATTCTACAGAATCTCCGAAAAAGAAAATCCAGCTTACTCTTTTTGTTT
TCTTCTTCACACGTGAGCTTTTCCGCCGGCATACGTTCCGTTCCGTGTCGTCTTGCATAAAATTTCCGAATCACATG
TTCGTAAAACAACCGGAAGTGCCCCGAATATAAAGTCAATTCTCACCGCTGTTGTAACTGGAGCTTTAAGGTGTTAT
CTAAGGAAGGATAAAAGAACTTAAACAACAACAAACAAAAAATTAAGTTACAATGCCAGCATTATTAAAAAGATTAT
TGTTTCAAGTGGGTCCTCATCCAAACGAAAGAACATTCACCTTATCCTCTGTTTCAACTGATGGGCACTATATTTCC
TTGAGACCATTTGTTAAGCCAAGCGGTGATGAGTTATCTTTTCCTTTCGAATGGGCCTTTGCCGGTACAAACGAAAC
AGTTAAAGCTAATGATCAAGGAAACGGTGTCGTTACTCAAGATTTCAATTTCTGGTTGGATACAAATGTGTACTTGA
ACGTTCCAAACACCCATCGTGGCGAAGTGAACACCACTTGGAAAAATTGGGATTCTGGTTGTGTCGAGGAAACAGGC
GCTGTTTACCCATTCGGTGCCGACAAAGAAAGCGTCTCTTTCAGAGAATTGTGGCAACCAGTTGACCCATCAAGAGA
AGATCTAGTCATCGTCTCACCAAACAATGAGAAGTTCTCGTCGAATGCTAGGTCAATTGTCCTCAAAGTTACTGACG
AAGCTTATGATGGTTTGGTTATTGTTATTGGTAGATGGATTCAAGGGTTTTTGTCCCAAAAGAATAATAACACTATT
GAAGGCTTGAACTTCATCAGATTACTTGAAAAAGATTCAGGTAAATCTGAGTTCTTATTAAGCTACGGTAAGGAAGT
AAACAAAATTCCACAAAGCTACGAAAATTTGAAGAAAGGTTCCACTGTAACCAGCAATGGGTTGAACTGGGAAGTTA
TTGAATATCACGCTTAATAAAGGAGAATAAATCGTTTTCTACTTTCTTCTGCTGCTATAATAAGCACCTATGGGATC
TATATAGTATTTTTATAACGATAGACTTTATAAAAGAAAATACCTAAGTGAAAATTTGGTGAATTTTGAGATAATTG
TTGGGATTCCATTTTTAATAAGGCAATAATATTAGGTATGTAGAATATACTAGAAGTTCTCCTCGAGGATTTAGGAA
TCCATAAAAGGGAATCTGCAATTCTACACAATTCTATAAATATTATTATCATCGTTTTATATGTTAATATTCATTGA
TCCTATTACATTATCAATCCTTGCGTTTCAGCTTCCACTAATTTAGATGACTATTTCTCATCATTTGCGTCATCTTC
TAACACCGTATATGATAATATACTAGTAACGTAAATACTAGTTAGTAGATGATAGTTGATTTTTATTCCAACACTAA
GAAATAATTTCGCCATTTCTTGAATGTATTTAAAGATATTTAATGCTATAATAGACATTTAAATCCAATTCTTCCAA
CATACAATGGGAGTTTGGCCGAGTGGTTTAAGGCGTCAGATTTAGGTGGATTTAACCTCTAAAATCTCTGATATCTT
CGGATGCAAGGGTTCGAATCCCTTAGCTCTCATTATTTTTTGCTTTTTCTCTTGAGGTCACATGATCGCAAAATGGC
AAATGGCACGTGAAGCTGTCGATATTGGGGAACTGTGGTGGTTGGCAAATGACTAATTAAGTTAGTCAAGGCGCCAT
CCTCATGAAAACTGTGTAACATAATAACCGAAGTGTCGAAAAGGTGGCACCTTGTCCAATTGAACACGCTCGATGAA
AAAAATAAGATATATATATAAGGTTAAGTAAAGCGTCTGTTAGAAAGGAAGTTTTTCCTTTTTCTTGCTCTCTTGTCTT
TTCATCTACTATTTCCTTCGTGTAATACAGGGTCGTCAGATACATAGATACAATTCTATTACCCCCATCCATACA**AT**
GCCATCTCATTTCGATACTGTTCAACTACACGCCGGCCAAGAGAACCCTGGTGACAATGCTCACAGATCCAGAGCTG
TACCAATTTACGCCACCACTTCTTATGTTTTCGAAAACTCTAAGCATGGTTCGCAATTGTTTGGTCTAGAAGTTCCA
GGTTACGTCTATTCCCGTTTCCAAAACCCAACCAGTAATGTTTTGGAAGAAAGAATTGCTGCTTTAGAAGGTGGTGC
TGCTGCTTTGGCTGTTTCCTCCGGTCAAGCCGCTCAAACCCTTGCCATCCAAGGTTTGGCACACACTGGTGACAACA
TCGTTTCCACTTCTTACTTATACGGTGGTACTTATAACCAGTTCAAAATCTCGTTCAAAAGATTTGGTATCGAGGCT
AGATTTGTTGAAGGTGACAATCCAGAAGAATTCGAAAAGGTCTTTGATGAAAGAACCAAGGCTGTTTATTTGGAAAC
CATTGGTAATCCAAAGTACAATGTTCCGGATTTTGAAAAAATTGTTGCAATTGCTCACAAACACGGTATTCCAGTTG
TCGTTGACAACACATTTGGTGCCGGTGGTTACTTCTGTCAGCCAATTAAATACGGTGCTGATATTGTAACACATTCT
GCTACCAAATGGATTGGTGGTCATGGTACTACTATCGGTGGTATTATTGTTGACTCTGGTAAGTTCCCATGGAAGGA

CTACCCAGAAAAGTTCCCTCAATTCTCTCAACCTGCCGAAGGATATCACGGTACTATCTACAATGAAGCCTACGGTA
ACTTGGCATACATCGTTCATGTTAGAACTGAACTATTAAGAGATTTGGGTCCATTGATGAACCCATTTGCCTCTTTC
TTGCTACTACAAGGTGTTGAAACATTATCTTTGAGAGCTGAAAGACACGGTGAAAATGCATTGAAGTTAGCCAAATG
GTTAGAACAATCCCCATACGTATCTTGGGTTTCATACCCTGGTTTAGCATCTCATTCTCATCATGAAAATGCTAAGA
AGTATCTATCTAACGGTTTCGGTGGTGTCTTATCTTTCGGTGTAAAAGACTTACCAAATGCCGACAAGGAAACTGAC
CCATTCAAACTTTCTGGTGCTCAAGTTGTTGACAATTTAAAGCTTGCCTCTAACTTGGCCAATGTTGGTGATGCCAA
GACCTTAGTCATTGCTCCATACTTCACTACCCACAAACAATTAAATGACAAAGAAAAGTTGGCATCTGGTGTTACCA
AGGACTTAATTCGTGTCTCTGTTGGTATCGAATTTATTGATGACATTATTGCAGACTTCCAGCAATCTTTTGAAACT
GTTTTCGCTGGCCAAAAACCATGAGTGTGCGTAATGAGTTGTAAAATTATGTATAAACCTACTTTCTCTCACAAGTA
CTATACTTTTATAAAACGAACTTTATTGAAATGAATATCCTTTTTTTCCCTTGTTACATGTCGTGACTCGTACTTTG
AACCTAAATTGTTCTAACATCAAAGAACAGTGTTAATTCGCAGTCGAGAAGAAAAATATGGTGAACAAGACTCATCT
ACTTCATGAGACTACTTTACGCCTCCTATAAAGCTGTCACACTGGATAAATTTATTGTAGGACCAAGTTACAAAAGA
GGATGATGGAGGTTTCTTTACAATAAAGAAGCACATGTGTGTTAACGTTTTTAGTATTTGCTTGTTATGTAAATCAG
GAAAACTTCGCGGGATTTGGTTGGATGCTACTTTCCATACAATAAATATTATAGATCTAAAAAGCCAAATTACAAGT
AAAGATTAGTAAAGCTGTTGGAATTCCATCGTTGATAAAAATGTTAGTTATTAAATATAAAAGTCAGAATAGGTGAA
CTTGGATTTAATTGTTGGCATTTCGTTGCTGCTAGAGGCCATAATATTAGATAGCCAGGACATACTAGTTCTCCTCG
TGGTATAGGAATCCATAAAATGGAATTGGTGATTCTATGTGATATATTCACATTCTTACTACATTATCAATCCTTGC
ACTTCAGCTTCCTCTAACCTCGATGACATCTTCTCATAACTTATGTCATCATCTAACGCCGTCTATTATAATATATT
GATAGTATAAGTATTAGTTGATAGACAATAGTGGATTTTTATTCCAACAGTGTCTTTGTTCGTCTCAGATATAGTCG
GATTGCCCTTTTAAGCAATCAATAGTGTTTTATTTGCAACAATGTCGTCATAGTTTAATATGTCCTATAAGATGTTA
ACTTGCTCAACATTCAACAAAGTTTGGTCTCTTGGCCCAGTTGGTTAAGGCACCGTGCTAATAACGCGGGGATCAGC
GGTTCGATCCCGCTAGAGACCATTTATTTTTAAATTCGACCGTCTGACAGACGGTGCACTTATTACTTAATTTTTTT
TTTTTTTTAATCCTCGTTAAACTGAGTACATAATCAGCAACAATATAATATAAAAGTTTTGCTTTTTTATGTACGTA
CAATAGGGGTACTTAAATAAATAAATAAACAATAAAACAATCAATAAATAAATAATATACGATCATAAATTACGAGA
TATATTTCATAGTCTTGACTTCTCCAGCCGAACGTCATTAAATTTAAGGCAGAAAAGATAAACAAGGGTACGGTGTA
CAGAAGACAAAATAAATAATAACCTTTAAAAGATTATAACAATGTTTTCATTATTTCTTCTCATCGGCCTTAATTTT
ATTTAAGGCAGAACCATATTTGAACCATTCAATTTGCTCATCGTTGAAAGTATGGGTCAACACAGCATCCCATGGCT
TACCATTCTTTGGATGAACTCTCATTGTTACAGGCTTACCTGGAGCCAATTCAGCTAGACCCAGAATATCGATTCTG
TCATCAGGGTTGATCTTGTCATAGTCAGCTGGGTTCTTGAAGTTCAATGGCAATAGACCTTGTTTTTTCAAGTTAGT
TTCATGGATACGAGCGAAAGACTTTGTGATGATAGCGAAACCGCCCAAGAATCTTGGTTCCAAAGCAGCGTGTTCAC
GAGAGGAACCTTCACCAAAGTTTTCATCACCAATAACAACCCACTTGATACCTTGGTCTCTGTAATCTCTAGCAGTG
TCTGGAACACCTTTGTATTCACCAGTATATACATTTTTAACACAGTTAGCCTTCTTGTTTTCAGCATTAATAGCACC
AATCATATAGTTATTAGAAATGTTTTCTAAATGACCTCTGTATTTCAACCATGGACCAGCCATAGAAATATGATCAG
TAGTTGTCTTACCGACGGCCTTAATCAAGATTGGCATGTCTTTAGCATCCTTACCATCCCAAGGTTTGAATGGTTTC
AACAGTTGTAGACGGTCTGAAG

***met15*Δ0 (from BY4741 background)**

*prMET15::**Added Sequence**::MET15(t)*

CCACAAAGCTACGAAAATTTGAAGAAAGGTTCCACTGTAACCAGCAATGGGTTGAACTGGGAAGTTATTGAATATCA
CGCTTAATAAAGGAGAATAAATCGTTTTCTACTTTCTTCTGCTGCTATAATAAGCACCTATGGGATCTATATAGTAT
TTTTATAACGATAGACTTTATAAAAGAAAATACCTAAGTGAAAATTTGGTGAATTTTGAGATAATTGTTGGGATTCC
ATTTTTAATAAGGCAATAATATTAGGTATGTAGAATATACTAGAAGTTCTCCTCGAGGATTTAGGAATCCATAAAAG
GGAATCTGCAATTCTACACAATTCTATAAATATTATTATCATCGTTTTATATGTTAATATTCATTGATCCTATTACA
TTATCAATCCTTGCGTTTCAGCTTCCACTAATTTAGATGACTATTTCTCATCATTTGCGTCATCTTCTAACACCGTA
TATGATAATATACTAGTAACGTAAATACTAGTTAGTAGATGATAGTTGATTTTTATTCCAACACTAAGAAATAATTT
CGCCATTTCTTGAATGTATTTAAAGATATTTAATGCTATAATAGACATTTAAATCCAATTCTTCCAACATACAATGG
GAGTTTGGCCGAGTGGTTTAAGGCGTCAGATTTAGGTGGATTTAACCTCTAAAATCTCTGATATCTTCGGATGCAAG
GGTTCGAATCCCTTAGCTCTCATTATTTTTTGCTTTTTCTCTTGAGGTCACATGATCGCAAAATGGCAAATGGCACG
TGAAGCTGTCGATATTGGGGAACTGTGGTGGTTGGCA**GGATC**CTCAGATATAGTCGGATTGCCCTTTTAAGCAATCA
ATAGTGTTTTATTTGCAACAATGTCGTCATAGTTTAATATGTCCTATAAGATGTTAACTTGCTCAACATTCAACAAA
GTTTGGTCTCTTGGCCCAGTTGGTTAAGGCACCGTGCTAATAACGCGGGGATCAGCGGTTCGATCCCGCTAGAGACC
ATTTATTTTTAAATTCGACCGTCTGACAGACGGTGCACTTATTACTTAATTTTTTTTTTTTTTTTAATCCTCGTTAAA
CTGAGTACATAATCAGCAACAATATAATATAAAAGTTTTGCTTTTTTATGTACGTACAATAGGGGTACTTAAATAAA
TAAATAAACAATAAAACAATCAATAAATAAATAATATACGATCATAAATTACGAGATATATTTCATAGTCTTGACTT
CTCCAGCCGAACGTCATTAAATTTAAGGCAGAAAAGATAAACAAGGGTACGGTGTACAGAAGACAAAATAAATAATA

ACCTTTAAAAGATTATAACAATGTTTTCATTATTTCTTCTCATCGGCCTTAATTTTATTTAAGGCAGAACCATATTT
GAACCATTCAATTTGCTCATCGTTGAAAGTATGGGTCAACACAGCATCCCATGGCTTACCATTCTTTGGATGAACTC
TCATTGTTACAGGCTTACCTGGAGCCAATTCAGCTAGACCCAGAATATCGATTCTGTCATCAGGGTTGATCTTGTCA
TAGTCAGCTGGGTTCTTGAAGTTCAATGGCAATAGACCTTGTTTTTTCAAGTTAGTTTCATGGATACGAGCGAAAGA
CTTTGTGATGATAGCGAAACCGCCCAAGAATCTTGGTTCCAAAGCAGCGTGTTCACGAGAGGAACCTTCACCAAAGT
TTTCATCACCAATAACAACCCACTTGATACCTTGGTCTCTGTAATCTCTAGCAGTGTCTGGAACACCTTTGTATTCA
CCAGTATATACATTTTTAACACAGTTAGCCTTCTTGTTTTCAGCATTAATAGCACCAATCATATAGTTATTAGAAAT
GTTTTCTAAATGACCTCTGTATTTCAACCATGGACCAGCCATAGAAATATGATCAGTAGTTGTCTTACCGACGGCCT
TAATCAAGATTGGCATGTCTTTAGCATCCTTACCATCCCAAGGTTTGAATGGTTTCAACAGTTGTAGACGGTCTGAA
G

Promoter region: 259 bp of immediate 5’ UTR deleted. “**GGATC”** sequence added in place of *MET15*. Terminator region: 809 bp of immediate 3’ UTR was deleted.

First Generation Yeast CRISPR Gene Drive

prHIS3-[u2]-prGAL1/10-SpCas9-NLS-ADH1(t)-prMX-Kan^{R}-MX(t)-[u2]-HIS3(t)
(Yeast Strain Name, GFY-2383)

GGGTCAGTTATTTCATCCAGATATAACCCGAGAGGAAACTTCTTAGCGTCTGTTTTCGTACCATAAGGCAGTTCATG
AGGTATATTTTCGTTATTGAAGCCCAGCTCGTGAATGCTTAATGCTGCTGAACTGGTGTCCATGTCGCCTAGGTACG
CAATCTCCACAGGCTGCAAAGGTTTTGTCTCAAGAGCAATGTTATTGTGCACCCCGTAATTGGTCAACAAGTTTAAT
CTGTGCTTGTCCACCAGCTCTGTCGTAACCTTCAGTTCATCGACTATCTGAAGAAATTTACTAGGAATAGTGCCATG
GTACAGCAACCGAGAATGGCAATTTCTACTCGGGTTCAGCAACGCTGCATAAACGCTGTTGGTGCCGTAGACATATT
CGAAGATAGGATTATCATTCATAAGTTTCAGAGCAATGTCCTTATTCTGGAACTTGGATTTATGGCTCTTTTGGTTT
AATTTCGCCTGATTCTTGATCTCCTTTAGCTTCTCGACGTGGGCCTTTTTCTTGCCATATGGATCCGCTGCACGGTC
CTGTTCCCTAGCATGTACGTGAGCGTATTTCCTTTTAAACCACGACGCTTTGTCTTCATTCAACGTTTCCCATTGTT
TTTTTCTACTATTGCTTTGCTGTGGGAAAAACTTATCGAAAGATGACGACTTTTTCTTAATTCTCGTTTTAAGAGCT
TGGTGAGCGCTAGGAGTCACTGCCAGGTATCGTTTGAACACGGCATTAGTCAGGGAAGTCATAACACAGTCCTTTCC
CGCAATTTTCTTTTTCTATTACTCTTGGCCTCCTCTAGTACACTCTATATTTTTTTATGCCTCGGTAATGATTTTCA
TTTTTTTTTTTCCACCTAGCGGATGACTCTTTTTTTTTCTTAGCGATTGGCATTATCACATAATGAATTATACATTA
TATAAAGTAATGTGATTTCTTCGAAGAATATACTAAAAAATGAGCAGGCAAGATAAACGAAGGCAAAG**GCTGTTCGT**
GTGCGCGTCCTGGGGACAGGTTATCAGCAACAACACAGTCATATCCATTCTCAATTAGCTCTACCACAGTGTGTGAA
CCAATGTATCCAGCACCACCTGTAACCAAAACAATTTTAGAAGTACTTTCACTTTGTAACTGAGCTGTCATTTATAT
TGAATTTTCAAAAATTCTTACTTTTTTTTTGGATGGACGCAAAGAAGTTTAATAATCATATTACATGGCATTACCAC
CATATACATATCCATATACATATCCATATCTAATCTTACTTATATGTTGTGGAAATGTAAAGAGCCCCATTATCTTA
GCCTAAAAAAACCTTCTCTTTGGAACTTTCAGTAATACGCTTAACTGCTCATTGCTATATTGAAGTACGGATTAGAA
GCCGCCGAGCGGGTGACAGCCCTCCGAAGGAAGACTCTCCTCCGTGCGTCCTCGTCTTCACCGGTCGCGTTCCTGAA
ACGCAGATGTGCCTCGCGCCGCACTGCTCCGAACAATAAAGATTCTACAATACTAGCTTTTATGGTTATGAAGAGGA
AAAATTGGCAGTAACCTGGCCCCACAAACCTTCAAATGAACGAATCAAATTAACAACCATAGGATGATAATGCGATT
AGTTTTTTAGCCTTATTTCTGGGGTAATTAATCAGCGAAGCGATGATTTTTGATCTATTAACAGATATATAAATGCA
AAAACTGCATAACCACTTTAACTAATACTTTCAACATTTTCGGTTTGTATTACTTCTTATTCAAATGTAATAAAAGT
ATCAACAAAAAATTGTTAATATACCTCTATACTTTAACGTCAAGGAGAAAAAACTATA**ATGGATAAGAAATACTCTA**
TCGGTTTGGATATTGGTACAAATTCAGTTGGTTGGGCAGTTATTACTGATGAATACAAGGTTCCATCTAAAAAGTTT
AAAGTTTTGGGTAACACTGATAGACATTCTATTAAGAAAAATTTGATTGGTGCTTTGTTATTTGATTCTGGTGAAAC
TGCTGAAGCAACAAGATTGAAAAGAACTGCAAGAAGAAGATACACAAGAAGAAAGAATAGAATCTGTTATTTGCAAG
AAATTTTCTCTAACGAAATGGCTAAGGTTGATGATTCTTTCTTTCATAGATTGGAAGAATCATTTTTAGTTGAAGAA
GATAAGAAACATGAAAGACATCCAATCTTCGGTAACATCGTTGATGAAGTTGCTTACCATGAAAAGTACCCAACAAT
CTATCATTTGAGAAAGAAATTGGTTGATTCAACTGATAAGGCAGATTTGAGATTGATATATTTGGCTTTAGCACATA
TGATCAAGTTTAGAGGTCATTTCTTGATCGAGGGTGACTTGAATCCAGATAATTCTGATGTTGATAAGTTGTTTATT
CAATTAGTTCAAACATATAATCAATTGTTTGAAGAAAATCCAATTAATGCTTCTGGTGTTGATGCTAAGGCAATCTT
GTCAGCAAGATTGTCTAAGTCAAGAAGATTGGAAAATTTGATCGCTCAATTACCAGGTGAAAAGAAAAATGGTTTGT
TCGGTAATTTGATCGCATTGTCTTTGGGTTTGACACCAAACTTCAAGTCAAACTTCGATTTGGCTGAAGATGCAAAG
TTGCAATTGTCTAAGGATACTTACGATGATGATTTGGATAATTTGTTGGCTCAAATTGGTGACCAATATGCAGATTT

GTTTTTGGCTGCTAAAAATTTGTCTGATGCTATCTTGTTGTCAGATATCTTGAGAGTTAACACTGAAATCACAAAGG
CTCCATTGTCTGCATCAATGATCAAGAGATACGATGAACATCATCAAGATTTGACTTTGTTGAAGGCATTGGTTAGA
CAACAATTACCAGAAAAGTACAAGGAAATTTTCTTTGATCAATCTAAAAATGGTTATGCTGGTTACATTGATGGTGG
TGCATCTCAAGAAGAATTCTACAAGTTTATTAAGCCAATCTTGGAAAAGATGGATGGTACAGAAGAATTGTTAGTTA
AATTGAACAGAGAAGATTTGTTAAGAAAACAAAGAACTTTCGATAACGGTTCTATCCCACATCAAATCCATTTGGGT
GAATTACATGCTATCTTGAGAAGACAAGAAGATTTCTACCCATTTTTAAAGGATAACAGAGAAAAGATTGAAAAGAT
TTTGACTTTTAGAATTCCATATTACGTTGGTCCATTAGCTCGTGGTAATTCTAGATTTGCATGGATGACTAGAAAGT
CAGAAGAAACTATCACACCATGGAATTTTGAAGAAGTTGTTGATAAAGGTGCTTCTGCACAATCTTTTATTGAAAGA
ATGACAAACTTCGATAAAAATTTGCCAAACGAAAAGGTTTTGCCAAAGCATTCATTGTTATATGAATACTTTACTGT
TTACAATGAATTGACAAAAGTTAAATATGTTACTGAGGGTATGAGAAAACCAGCATTTTTGTCTGGTGAACAAAAGA
AAGCAATCGTTGATTTGTTGTTTAAAACTAACAGAAAGGTTACAGTTAAACAATTGAAAGAAGATTACTTTAAGAAA
ATTGAATGTTTTGATTCTGTTGAAATTTCAGGTGTTGAAGATAGATTCAATGCTTCATTAGGTACTTACCATGATTT
GTTGAAGATTATTAAGGATAAAGATTTCTTGGATAATGAAGAAAATGAAGATATTTTAGAAGATATTGTTTTAACTT
TGACATTATTTGAAGATAGAGAAATGATCGAAGAAAGATTGAAGACATACGCTCATTTGTTCGATGATAAAGTTATG
AAGCAATTGAAGAGAAGAAGATACACTGGTTGGGGTAGATTGTCTAGAAAGTTGATTAATGGTATCAGAGATAAGCA
ATCTGGTAAAACAATCTTGGATTTCTTGAAGTCAGATGGTTTCGCAAACAGAAACTTCATGCAATTGATTCATGATG
ATTCATTGACTTTTAAAGAAGATATCCAAAAAGCTCAAGTTTCTGGTCAGGGTGACTCATTGCATGAACATATTGCT
AATTTGGCAGGTTCTCCAGCTATTAAGAAAGGTATCTTGCAAACAGTTAAGGTTGTTGATGAATTAGTTAAAGTTAT
GGGTAGACATAAGCCAGAAAACATCGTTATCGAAATGGCTAGAGAAAACCAAACTACACAAAAGGGTCAAAAGAATT
CAAGAGAAAGAATGAAGAGAATCGAAGAAGGTATTAAAGAATTGGGTTCTCAAATCTTGAAGGAACATCCAGTTGAA
AACACTCAATTGCAAAACGAAAAGTTGTACTTATACTACTTACAAAACGGTAGAGATATGTACGTTGATCAAGAATT
AGATATCAACAGATTGTCAGATTACGATGTTGATCATATCGTTCCACAATCATTTTTGAAGGATGATTCAATCGATA
ATAAGGTTTTGACAAGATCTGATAAGAACCGTGGTAAATCTGATAATGTTCCATCAGAAGAAGTTGTTAAGAAAATG
AAGAACTACTGGAGACAATTGTTAAATGCTAAGTTGATCACTCAAAGAAAGTTCGATAATTTGACAAAAGCTGAAAG
AGGTGGTTTGTCAGAATTAGATAAAGCAGGTTTTATTAAGAGACAATTAGTTGAAACTAGACAAATCACAAAGCATG
TTGCACAAATCTTGGATTCTAGAATGAACACTAAATATGATGAAAATGATAAATTAATTAGAGAAGTTAAAGTTATT
ACATTAAAATCTAAATTGGTTTCAGATTTTAGAAAAGATTTTCAATTCTACAAAGTTAGAGAAATTAATAACTATCA
TCATGCTCATGATGCATACTTGAATGCTGTTGTTGGTACTGCATTGATTAAGAAATACCCAAAGTTGGAATCTGAAT
TCGTTTACGGTGACTACAAGGTTTACGATGTTAGAAAGATGATCGCTAAGTCAGAACAAGAAATCGGTAAAGCTACA
GCAAAGTATTTCTTTTATTCTAACATCATGAATTTCTTTAAAACTGAAATTACATTAGCTAACGGTGAAATCAGAAA
AAGACCATTGATCGAAACTAATGGTGAAACAGGTGAAATTGTTTGGGATAAAGGTAGAGATTTCGCAACTGTTAGAA
AGGTTTTGTCAATGCCACAAGTTAACATCGTTAAGAAAACTGAAGTTCAAACAGGTGGTTTTTCTAAGGAATCAATC
TTGCCAAAGAGAAACTCTGATAAGTTGATTGCTAGAAAGAAAGATTGGGATCCAAAGAAATATGGTGGTTTTGATTC
TCCAACTGTTGCTTACTCAGTTTTAGTTGTTGCAAAGGTTGAAAAGGGTAAATCTAAGAAATTGAAATCAGTTAAAG
AATTGTTAGGTATCACAATCATGGAAAGATCTTCATTCGAAAAGAATCCAATCGATTTCTTGGAAGCAAAGGGTTAC
AAGGAAGTTAAGAAAGATTTGATTATTAAGTTGCCAAAGTACTCTTTGTTCGAATTAGAAAACGGTAGAAAAAGAAT
GTTAGCTTCAGCTGGTGAATTGCAAAAGGGTAATGAATTGGCTTTGCCATCTAAGTACGTTAATTTCTTGTATTTGG
CATCTCATTACGAAAAGTTGAAGGGTTCACCAGAAGATAATGAACAAAAACAATTGTTCGTTGAACAACATAAGCAT
TATTTGGATGAAATTATTGAACAAATTTCTGAATTTTCAAAAAGAGTTATTTTGGCTGATGCAAATTTGGATAAGGT
TTTGTCTGCTTACAATAAGCATAGAGATAAGCCAATCAGAGAACAAGCAGAAAACATCATCCATTTGTTTACTTTGA
CAAATTTGGGTGCTCCAGCTGCTTTTAAATACTTCGATACTACAATCGATAGAAAAAGATACACTTCTACAAAGGAA
GTTTTGGATGCAACATTGATCCATCAATCAATCACTGGTTTGTATGAAACAAGAATTGATTTGTCTCAATTGGGTGG
TGACTCTAGGGCAGACCCAAAGAAAAAGAGGAAAGTATAAGGCGCGCCATTCTAAATAAGCGAATTTCTTATGATTT
ATGATTTTTATTATTAAATAAGTTATAAAAAAAATAAGTGTATACAAATTTTAAAGTGACTCTTAGGTTTTAAAACG
AAAATTCTTATTCTTGAGTAACTCTTTCCTGTAGGTCAGGTTGCTTTCTCAGGTATAGTATGAGGTCGCTCTTATTG
ACCACACCTCTACCGGCAGATCCGCTAGGGATAACAGGGTAATATAGATCTGTTTAGCTTGCCTCGTCCCCGCCGGG
TCACCCGGCCAGCGACATGGAGGCCCAGAATACCCTCCTTGACAGTCTTGACGTGCGCAGCTCAGGGGCATGATGTG
ACTGTCGCCCGTACATTTAGCCCATACATCCCCATGTATAATCATTTGCATCCATACATTTTGATGGCCGCACGGCG
CGAAGCAAAAATTACGGCTCCTCGCTGCAGACCTGCGAGCAGGGAAACGCTCCCCTCACAGACGCGTTGAATTGTCC
CCACGCCGCGCCCCTGTAGAGAAATATAAAAGGTTAGGATTTGCCACTGAGGTTCTTCTTTCATATACTTCCTTTTA
AAATCTTGCTAGGATACAGTTCTCACATCACATCCGAACATAAACAACCATGGGTAAGGAAAAGACTCACGTTTCGA
GGCCGCGATTAAATTCCAACATGGATGCTGATTTATATGGGTATAAATGGGCTCGCGATAATGTCGGGCAATCAGGT
GCGACAATCTATCGATTGTATGGGAAGCCCGATGCGCCAGAGTTGTTTCTGAAACATGGCAAAGGTAGCGTTGCCAA
TGATGTTACAGATGAGATGGTCAGACTAAACTGGCTGACGGAATTTATGCCTCTTCCGACCATCAAGCATTTTATCC
GTACTCCTGATGATGCATGGTTACTCACCACTGCGATCCCCGGCAAAACAGCATTCCAGGTATTAGAAGAATATCCT
GATTCAGGTGAAAATATTGTTGATGCGCTGGCAGTGTTCCTGCGCCGGTTGCATTCGATTCCTGTTTGTAATTGTCC
TTTTAACAGCGATCGCGTATTTCGTCTCGCTCAGGCGCAATCACGAATGAATAACGGTTTGGTTGATGCGAGTGATT

TTGATGACGAGCGTAATGGCTGGCCTGTTGAACAAGTCTGGAAAGAAATGCATAAGCTTTTGCCATTCTCACCGGAT
TCAGTCGTCACTCATGGTGATTTCTCACTTGATAACCTTATTTTTGACGAGGGGAAATTAATAGGTTGTATTGATGT
TGGACGAGTCGGAATCGCAGACCGATACCAGGATCTTGCCATCCTATGGAACTGCCTCGGTGAGTTTTCTCCTTCAT
TACAGAAACGGCTTTTTCAAAAATATGGTATTGATAATCCTGATATGAATAAATTGCAGTTTCATTTGATGCTCGAT
GAGTTTTTCTAATCAGTACTGACAATAAAAAGATTCTTGTTTTCAAGAACTTGTCATTTGTATAGTTTTTTTATATT
GTAGTTGTTCTATTTTAATCAAATGTTAGCGTGATTTATATTTTTTTTCGCCTCGACATCATCTGCCCAGATGCGAA
GTTAAGTGCGCAGAAAGTAATATCATGCGTCAATCGTATGTGAATGCTGGTCGCTATACTGCTGTCGATTCGATACT
AACGCCGCCATCCAGTGCTGTTCGTGTGCGCGTCCTGGGTGACACCGATTATTTAAAGCTGCAGCATACGATATATA
TACATGTGTATATATGTATACCTATGAATGTCAGTAAGTATGTATACGAACAGTATGATACTGAAGATGACAAGGTA
ATGCATCATTCTATACGTGTCATTCTGAACGAGGCGCGCTTTCCTTTTTTCTTTTTGCTTTTTCTTTTTTTTTCTCT
TGAACTCGAGAAAAAAAATATAAAAGAGATGGAGGAACGGGAAAAAGTTAGTTGTGGTGATAGGTGGCAAGTGGTAT
TCCGTAAGAACAACAAGAAAAGCATTTCATATTATGGCTGAACTGAGCGAACAAGTGCAAAATTTAAGCATCAACGA
CAACAACGAGAATGGTTATGTTCCTCCTCACTTAAGAGGAAAACCAAGAAGTGCCAGAAATAACAGTAGCAACTACA
ATAACAACAACGGCGGCTACAACGGTGGCCGTGGCGGTGGCAGCTTCTTTAGCAACAACCGTCGTGGTGGTTACGGC
AACGGTGGTTTCTTCGGTGGAAACAACGGTGGCAGCAGATCTAACGGCCGTTCTGGTGGTAGATGGATCGATGGCAA
ACATGTCCCAGCTCCAAGAAACGAAAAGGCCGAGATCGCCATATTTGGTGTCCCCGAGGATCCAAATTTCCAATCTT
CTGGTATTAACTTCGATAACTACGATGATATTCCAGTGGACGCCTCTGGTAAGGATGTTCCTGAACCAATCACAGAA
TTTACCTCACCTCCATTGGACGGATTGTTATTGGAAAACATCAAATTGGCCCGTTTCACCAAGCCAACACCTGTGCA
AAAATACTCCGTCCCTATCGTTGCCAACGGCAGAGATTTGATGGCCTGTGCGCAGACCGGTTCTGGTAAGACTGGTG
GGTTTTTATTCCCAGTGTTGTCCGAATCATTTAAGACTGGACCATCTCCTCAACCAGAGTCTCAAGGCTCCTTTTAC
CAAAGAAAGGCCTACCCAACTGCTGTCATTA

Second Generation Yeast CRISPR Gene Drive

prHIS3-[u2’]-prGAL1/10-SpCas9-NLS-ADH1(t)-sgRNA(u1)-prMX-CaURA3-MX(t)-[u2’]-HIS3(t)
(Yeast Strain Names, GFY-4325/GFY-4326)

GGGTCAGTTATTTCATCCAGATATAACCCGAGAGGAAACTTCTTAGCGTCTGTTTTCGTACCATAAGGCAGTTCATG
AGGTATATTTTCGTTATTGAAGCCCAGCTCGTGAATGCTTAATGCTGCTGAACTGGTGTCCATGTCGCCTAGGTACG
CAATCTCCACAGGCTGCAAAGGTTTTGTCTCAAGAGCAATGTTATTGTGCACCCCGTAATTGGTCAACAAGTTTAAT
CTGTGCTTGTCCACCAGCTCTGTCGTAACCTTCAGTTCATCGACTATCTGAAGAAATTTACTAGGAATAGTGCCATG
GTACAGCAACCGAGAATGGCAATTTCTACTCGGGTTCAGCAACGCTGCATAAACGCTGTTGGTGCCGTAGACATATT
CGAAGATAGGATTATCATTCATAAGTTTCAGAGCAATGTCCTTATTCTGGAACTTGGATTTATGGCTCTTTTGGTTT
AATTTCGCCTGATTCTTGATCTCCTTTAGCTTCTCGACGTGGGCCTTTTTCTTGCCATATGGATCCGCTGCACGGTC
CTGTTCCCTAGCATGTACGTGAGCGTATTTCCTTTTAAACCACGACGCTTTGTCTTCATTCAACGTTTCCCATTGTT
TTTTTCTACTATTGCTTTGCTGTGGGAAAAACTTATCGAAAGATGACGACTTTTTCTTAATTCTCGTTTTAAGAGCT
TGGTGAGCGCTAGGAGTCACTGCCAGGTATCGTTTGAACACGGCATTAGTCAGGGAAGTCATAACACAGTCCTTTCC
CGCAATTTTCTTTTTCTATTACTCTTGGCCTCCTCTAGTACACTCTATATTTTTTTATGCCTCGGTAATGATTTTCA
TTTTTTTTTTTCCACCTAGCGGATGACTCTTTTTTTTTCTTAGCGATTGGCATTATCACATAATGAATTATACATTA
TATAAAGTAATGTGATTTCTTCGAAGAATATACTAAAAAATGAGCAGGCAAGATAAACGAAGGCAAAGTTTTCGCTG
TTCGTGTGCGCGTCCTGGGAGTGACAGGTTATCAGCAACAACACAGTCATATCCATTCTCAATTAGCTCTACCACAG
TGTGTGAACCAATGTATCCAGCACCACCTGTAACCAAAACAATTTTAGAAGTACTTTCACTTTGTAACTGAGCTGTC
ATTTATATTGAATTTTCAAAAATTCTTACTTTTTTTTTGGATGGACGCAAAGAAGTTTAATAATCATATTACATGGC
ATTACCACCATATACATATCCATATACATATCCATATCTAATCTTACTTATATGTTGTGGAAATGTAAAGAGCCCCA
TTATCTTAGCCTAAAAAAACCTTCTCTTTGGAACTTTCAGTAATACGCTTAACTGCTCATTGCTATATTGAAGTACG
GATTAGAAGCCGCCGAGCGGGTGACAGCCCTCCGAAGGAAGACTCTCCTCCGTGCGTCCTCGTCTTCACCGGTCGCG
TTCCTGAAACGCAGATGTGCCTCGCGCCGCACTGCTCCGAACAATAAAGATTCTACAATACTAGCTTTTATGGTTAT
GAAGAGGAAAAATTGGCAGTAACCTGGCCCCACAAACCTTCAAATGAACGAATCAAATTAACAACCATAGGATGATA
ATGCGATTAGTTTTTTAGCCTTATTTCTGGGGTAATTAATCAGCGAAGCGATGATTTTTGATCTATTAACAGATATA
TAAATGCAAAAACTGCATAACCACTTTAACTAATACTTTCAACATTTTCGGTTTGTATTACTTCTTATTCAAATGTA
ATAAAAGTATCAACAAAAAAATTGTTAATATACCTCTATACTTTAACGTCAAGGAGAAAAAACTATAATGGATAAGAA
ATACTCTATCGGTTTGGATATTGGTACAAATTCAGTTGGTTGGGCAGTTATTACTGATGAATACAAGGTTCCATCTA
AAAAGTTTAAAGTTTTGGGTAACACTGATAGACATTCTATTAAGAAAAATTTGATTGGTGCTTTGTTATTTGATTCT
GGTGAAACTGCTGAAGCAACAAGATTGAAAAGAACTGCAAGAAGAAGATACACAAGAAGAAAGAATAGAATCTGTTA
TTTGCAAGAAATTTTCTCTAACGAAATGGCTAAGGTTGATGATTCTTTCTTTCATAGATTGGAAGAATCATTTTTAG

TTGAAGAAGATAAGAAACATGAAAGACATCCAATCTTCGGTAACATCGTTGATGAAGTTGCTTACCATGAAAAGTAC
CCAACAATCTATCATTTGAGAAAGAAATTGGTTGATTCAACTGATAAGGCAGATTTGAGATTGATATATTTGGCTTT
AGCACATATGATCAAGTTTAGAGGTCATTTCTTGATCGAGGGTGACTTGAATCCAGATAATTCTGATGTTGATAAGT
TGTTTATTCAATTAGTTCAAACATATAATCAATTGTTTGAAGAAAATCCAATTAATGCTTCTGGTGTTGATGCTAAG
GCAATCTTGTCAGCAAGATTGTCTAAGTCAAGAAGATTGGAAAATTTGATCGCTCAATTACCAGGTGAAAAGAAAAA
TGGTTTGTTCGGTAATTTGATCGCATTGTCTTTGGGTTTGACACCAAACTTCAAGTCAAACTTCGATTTGGCTGAAG
ATGCAAAGTTGCAATTGTCTAAGGATACTTACGATGATGATTTGGATAATTTGTTGGCTCAAATTGGTGACCAATAT
GCAGATTTGTTTTTGGCTGCTAAAAATTTGTCTGATGCTATCTTGTTGTCAGATATCTTGAGAGTTAACACTGAAAT
CACAAAGGCTCCATTGTCTGCATCAATGATCAAGAGATACGATGAACATCATCAAGATTTGACTTTGTTGAAGGCAT
TGGTTAGACAACAATTACCAGAAAAGTACAAGGAAATTTTCTTTGATCAATCTAAAAATGGTTATGCTGGTTACATT
GATGGTGGTGCATCTCAAGAAGAATTCTACAAGTTTATTAAGCCAATCTTGGAAAAGATGGATGGTACAGAAGAATT
GTTAGTTAAATTGAACAGAGAAGATTTGTTAAGAAAACAAAGAACTTTCGATAACGGTTCTATCCCACATCAAATCC
ATTTGGGTGAATTACATGCTATCTTGAGAAGACAAGAAGATTTCTACCCATTTTTAAAGGATAACAGAGAAAAGATT
GAAAAGATTTTGACTTTTAGAATTCCATATTACGTTGGTCCATTAGCTCGTGGTAATTCTAGATTTGCATGGATGAC
TAGAAAGTCAGAAGAAACTATCACACCATGGAATTTTGAAGAAGTTGTTGATAAAGGTGCTTCTGCACAATCTTTTA
TTGAAAGAATGACAAACTTCGATAAAAATTTGCCAAACGAAAAGGTTTTGCCAAAGCATTCATTGTTATATGAATAC
TTTACTGTTTACAATGAATTGACAAAAGTTAAATATGTTACTGAGGGTATGAGAAAACCAGCATTTTTGTCTGGTGA
ACAAAAGAAAGCAATCGTTGATTTGTTGTTTAAAACTAACAGAAAGGTTACAGTTAAACAATTGAAAGAAGATTACT
TTAAGAAAATTGAATGTTTTGATTCTGTTGAAATTTCAGGTGTTGAAGATAGATTCAATGCTTCATTAGGTACTTAC
CATGATTTGTTGAAGATTATTAAGGATAAAGATTTCTTGGATAATGAAGAAAATGAAGATATTTTAGAAGATATTGT
TTTAACTTTGACATTATTTGAAGATAGAGAAATGATCGAAGAAAGATTGAAGACATACGCTCATTTGTTCGATGATA
AAGTTATGAAGCAATTGAAGAGAAGAAGATACACTGGTTGGGGTAGATTGTCTAGAAAGTTGATTAATGGTATCAGA
GATAAGCAATCTGGTAAAACAATCTTGGATTTCTTGAAGTCAGATGGTTTCGCAAACAGAAACTTCATGCAATTGAT
TCATGATGATTCATTGACTTTTAAAGAAGATATCCAAAAAGCTCAAGTTTCTGGTCAGGGTGACTCATTGCATGAAC
ATATTGCTAATTTGGCAGGTTCTCCAGCTATTAAGAAAGGTATCTTGCAAACAGTTAAGGTTGTTGATGAATTAGTT
AAAGTTATGGGTAGACATAAGCCAGAAAACATCGTTATCGAAATGGCTAGAGAAAACCAAACTACACAAAAGGGTCA
AAAGAATTCAAGAGAAAGAATGAAGAGAATCGAAGAAGGTATTAAAGAATTGGGTTCTCAAATCTTGAAGGAACATC
CAGTTGAAAACACTCAATTGCAAAACGAAAAGTTGTACTTATACTACTTACAAAACGGTAGAGATATGTACGTTGAT
CAAGAATTAGATATCAACAGATTGTCAGATTACGATGTTGATCATATCGTTCCACAATCATTTTTGAAGGATGATTC
AATCGATAATAAGGTTTTGACAAGATCTGATAAGAACCGTGGTAAATCTGATAATGTTCCATCAGAAGAAGTTGTTA
AGAAAATGAAGAACTACTGGAGACAATTGTTAAATGCTAAGTTGATCACTCAAAGAAAGTTCGATAATTTGACAAAA
GCTGAAAGAGGTGGTTTGTCAGAATTAGATAAAGCAGGTTTTATTAAGAGACAATTAGTTGAAACTAGACAAATCAC
AAAGCATGTTGCACAAATCTTGGATTCTAGAATGAACACTAAATATGATGAAAATGATAAATTAATTAGAGAAGTTA
AAGTTATTACATTAAAATCTAAATTGGTTTCAGATTTTAGAAAAGATTTTCAATTCTACAAAGTTAGAGAAATTAAT
AACTATCATCATGCTCATGATGCATACTTGAATGCTGTTGTTGGTACTGCATTGATTAAGAAATACCCAAAGTTGGA
ATCTGAATTCGTTTACGGTGACTACAAGGTTTACGATGTTAGAAAGATGATCGCTAAGTCAGAACAAGAAATCGGTA
AAGCTACAGCAAAGTATTTCTTTTATTCTAACATCATGAATTTCTTTAAAACTGAAATTACATTAGCTAACGGTGAA
ATCAGAAAAAGACCATTGATCGAAACTAATGGTGAAACAGGTGAAATTGTTTGGGATAAAGGTAGAGATTTCGCAAC
TGTTAGAAAGGTTTTGTCAATGCCACAAGTTAACATCGTTAAGAAAACTGAAGTTCAAACAGGTGGTTTTTCTAAGG
AATCAATCTTGCCAAAGAGAAACTCTGATAAGTTGATTGCTAGAAAGAAAGATTGGGATCCAAAGAAATATGGTGGT
TTTGATTCTCCAACTGTTGCTTACTCAGTTTTAGTTGTTGCAAAGGTTGAAAAGGGTAAATCTAAGAAATTGAAATC
AGTTAAAGAATTGTTAGGTATCACAATCATGGAAAGATCTTCATTCGAAAAGAATCCAATCGATTTCTTGGAAGCAA
AGGGTTACAAGGAAGTTAAGAAAGATTTGATTATTAAGTTGCCAAAGTACTCTTTGTTCGAATTAGAAAACGGTAGA
AAAAGAATGTTAGCTTCAGCTGGTGAATTGCAAAAGGGTAATGAATTGGCTTTGCCATCTAAGTACGTTAATTTCTT
GTATTTGGCATCTCATTACGAAAAGTTGAAGGGTTCACCAGAAGATAATGAACAAAAACAATTGTTCGTTGAACAAC
ATAAGCATTATTTGGATGAAATTATTGAACAAATTTCTGAATTTTCAAAAAGAGTTATTTTGGCTGATGCAAATTTG
GATAAGGTTTTGTCTGCTTACAATAAGCATAGAGATAAGCCAATCAGAGAACAAGCAGAAAACATCATCCATTTGTT
TACTTTGACAAATTTGGGTGCTCCAGCTGCTTTTAAATACTTCGATACTACAATCGATAGAAAAAGATACACTTCTA
CAAAGGAAGTTTTGGATGCAACATTGATCCATCAATCAATCACTGGTTTGTATGAAACAAGAATTGATTTGTCTCAA
TTGGGTGGTGACTCTAGGGCAGACCCAAAGAAAAAGAGGAAAGTATAAGGCGCGCCATTCTAAATAAGCGAATTTCT
TATGATTTATGATTTTTATTATTAAATAAGTTATAAAAAAAATAAGTGTATACAAATTTTAAAGTGACTCTTAGGTT
TTAAAACGAAAATTCTTATTCTTGAGTAACTCTTTCCTGTAGGTCAGGTTGCTTTCTCAGGTATAGTATGAGGTCGC
TCTTATTGACCACACCTCTACCGGCAGATCCGCTAGGGATAACAGGGTAATATAGGGAACAAAAGCTGGAGCTTCTT
TGAAAAGATAATGTATGATTATGCTTTCACTCATATTTATACAGAAACTTGATGTTTTCTTTCGAGTATATACAAGG
TGATTACATGTACGTTTGAAGTACAACTCTAGATTTTGTAGTGCCCTCTTGGGCTAGCGGTAAAGGTGCGCATTTTT
TCACACCCTACAATGTTCTGTTCAAAAGATTTTGGTCAAACGCTGTAGAAGTGAAAGTTGGTGCGCATGTTTCGGCG
TTCGAAACTTCTCCGCAGTGAAAGATAAATGATCCGGTGGACTTCGGCTACGTAGTTTTAGAGCTAGAAATAGCAAG

TTAAAATAAGGCTAGTCCGTTATCAACTTGAAAAAGTGGCACCGAGTCGGTGGTGC**TTTTTTTGTTTTTTATGTCT**T
CGAGTCATGTAATTAGTTATGTGTTTAGCTTGCCTCGTCCCCGCCGGGTCACCCGGCCAGCGACATGGAGGCCCAGA
ATACCCTCCTTGACAGTCTTGACGTGCGCAGCTCAGGGGCATGATGTGACTGTCGCCCGTACATTTAGCCCATACAT
CCCCATGTATAATCATTTGCATCCATACATTTTGATGGCCGCACGGCGCGAAGCAAAAATTACGGCTCCTCGCTGCA
GACCTGCGAGCAGGGAAACGCTCCCCTCACAGACGCGTTGAATTGTCCCCACGCCGCGCCCCTGTAGAGAAATATAA
AAGGTTAGGATTTGCCACTGAGGTTCTTCTTTCATATACTTCCTTTTAAAATCTTGCTAGGATACAGTTCTCACATC
ACATCCGAACATAAACAACC**ATGACAGTCAACACTAAGACCTATAGTGAGAGAGCAGAAACTCATGCCTCACCAGTA**
GCACAACGATTATTTCGATTAATGGAACTGAAGAAAACCAATTTATGTGCATCAATTGATGTTGATACCACTAAGGA
ATTCCTTGAATTAATTGATAAATTGGGTCCTTATGTATGCTTAATCAAGACTCATATTGATATAATCAATGATTTTT
CCTATGAATCCACTATTGAACCATTATTAGAACTTTCACGTAAACATCAATTTATGATTTTTGAAGATAGAAAATTT
GCTGATATTGGTAATACCGTGAAGAAACAATATATTGGTGGAGTTTATAAAATTAGTAGTTGGGCAGATATTACTAA
TGCTCATGGTGTCACTGGGAATGGAGTAGTTGAAGGATTAAAACAGGGAGCTAAAGAAACCACCACCAACCAAGAGC
CAAGAGGGTTATTGATGTTAGCTGAATTATCATCAGTGGGATCATTAGCATATGGAGAATATTCTCAAAAAACTGTT
GAAATTGCTAAATCCGATAAGGAATTTGTTATTGGATTTATTGCCCAACGTGATATGGGTGGACAAGAAGAAGGATT
TGATTGGCTTATTATGACACCTGGAGTTGGATTAGATGATAAAGGTGATGGATTAGGACAACAATATAGAACTGTTG
ATGAAGTTGTTAGCACTGGAACTGATATTATCATTGTTGGTAGAGGATTGTTTGGTAAAGGAAGAGATCCAGATATT
GAAGGTAAAAGGTATAGAGATGCTGGTTGGAATGCTTATTTGAAAAAGACTGGCCAATTATAATCAGTACTGACAAT
AAAAAGATTCTTGTTTTCAAGAACTTGTCATTTGTATAGTTTTTTTATATTGTAGTTGTTCTATTTTAATCAAATGT
TAGCGTGATTTATATTTTTTTTCGCCTCGACATCATCTGCCCAGATGCGAAGTTAAGTGCGCAGAAAGTAATATCAT
GCGTCAATCGTATGTGAATGCTGGTCGCTATACTGCTGTCGATTCGATACTAACGCCGCCATCCAGT**TTTCGCTGTT**
CGTGTGCGCGTCCTGGGAGTGACACCGATTATTTAAAGCTGCAGCATACGATATATATACATGTGTATATATGTATA
CCTATGAATGTCAGTAAGTATGTATACGAACAGTATGATACTGAAGATGACAAGGTAATGCATCATTCTATACGTGT
CATTCTGAACGAGGCGCGCTTTCCTTTTTTCTTTTTGCTTTTTCTTTTTTTTTCTCTTGAACTCGAGAAAAAAAATA
TAAAAGAGATGGAGGAACGGGAAAAAGTTAGTTGTGGTGATAGGTGGCAAGTGGTATTCCGTAAGAACAACAAGAAA
AGCATTTCATATTATGGCTGAACTGAGCGAACAAGTGCAAAATTTAAGCATCAACGACAACAACGAGAATGGTTATG
TTCCTCCTCACTTAAGAGGAAAACCAAGAAGTGCCAGAAATAACAGTAGCAACTACAATAACAACAACGGCGGCTAC
AACGGTGGCCGTGGCGGTGGCAGCTTCTTTAGCAACAACCGTCGTGGTGGTTACGGCAACGGTGGTTTCTTCGGTGG
AAACAACGGTGGCAGCAGATCTAACGGCCGTTCTGGTGGTAGATGGATCGATGGCAAACATGTCCCAGCTCCAAGAA
ACGAAAAGGCCGAGATCGCCATATTTGGTGTCCCCGAGGATCCAAATTTCCAATCTTCTGGTATTAACTTCGATAAC
TACGATGATATTCCAGTGGACGCCTCTGGTAAGGATGTTCCTGAACCAATCACAGAATTTACCTCACCTCCATTGGA
CGGATTGTTATTGGAAAACATCAAATTGGCCCGTTTCACCAAGCCAACACCTGTGCAAAAATACTCCGTCCCTATCG
TTGCCAACGGCAGAGATTTGATGGCCTGTGCGCAGACCGGTTCTGGTAAGACTGGTGGGTTTTTATTCCCAGTGTTG
TCCGAATCATTTAAGACTGGACCATCTCCTCAACCAGAGTCTCAAGGCTCCTTTTACCAAAGAAAGGCCTACCCAAC
TGCTGTCATTA

Second Generation Target Strain

prHIS3-[u1’]-prCDC12-mCherry-SHS1(t)-prCCW12-SpHIS5-MX(t)-[u1’]-HIS3(t)
(Yeast Strain Name, GFY-3733)

GGGTCAGTTATTTCATCCAGATATAACCCGAGAGGAAACTTCTTAGCGTCTGTTTTCGTACCATAAGGCAGTTCATG
AGGTATATTTTCGTTATTGAAGCCCAGCTCGTGAATGCTTAATGCTGCTGAACTGGTGTCCATGTCGCCTAGGTACG
CAATCTCCACAGGCTGCAAAGGTTTTGTCTCAAGAGCAATGTTATTGTGCACCCCGTAATTGGTCAACAAGTTTAAT
CTGTGCTTGTCCACCAGCTCTGTCGTAACCTTCAGTTCATCGACTATCTGAAGAAATTTACTAGGAATAGTGCCATG
GTACAGCAACCGAGAATGGCAATTTCTACTCGGGTTCAGCAACGCTGCATAAACGCTGTTGGTGCCGTAGACATATT
CGAAGATAGGATTATCATTCATAAGTTTCAGAGCAATGTCCTTATTCTGGAACTTGGATTTATGGCTCTTTTGGTTT
AATTTCGCCTGATTCTTGATCTCCTTTAGCTTCTCGACGTGGGCCTTTTTCTTGCCATATGGATCCGCTGCACGGTC
CTGTTCCCTAGCATGTACGTGAGCGTATTTCCTTTTAAACCACGACGCTTTGTCTTCATTCAACGTTTCCCATTGTT
TTTTTCTACTATTGCTTTGCTGTGGGAAAAACTTATCGAAAGATGACGACTTTTTCTTAATTCTCGTTTTAAGAGCT
TGGTGAGCGCTAGGAGTCACTGCCAGGTATCGTTTGAACACGGCATTAGTCAGGGAAGTCATAACACAGTCCTTTCC
CGCAATTTTCTTTTTCTATTACTCTTGGCCTCCTCTAGTACACTCTATATTTTTTTATGCCTCGGTAATGATTTTCA
TTTTTTTTTTTCCACCTAGCGGATGACTCTTTTTTTTTCTTAGCGATTGGCATTATCACATAATGAATTATACATTA
TATAAAGTAATGTGATTTCTTCGAAGAATATACTAAAAAATGAGCAGGCAAGATAAACGAAGGCAAAGT**TTTCCGGT**
GGACTTCGGCTACGTAGGGAGTGGGGCAGCGCCCTGTTTTTCATTAATGTAGTCAGCAATGTCAAGATTCAACGCCA
AGTCTGGTTCAGCAAGTGACATTCTGCAAGCTCTTTGAATCTTCCTCAAAAGAGGATTGCCCAAGGCTTGAGGTTTC
CTGACGGGCAACTCAGACAAATATATGCTATGTGAGTGCGGATGGGACATGATGCAGTATCACGATTAGCAATTCAG

CTATGAGTTATGTTGCTCTTTGTTTTGTTTATGGAAATTGTCCTATGGTAAGTCTCTTTTTTTTGCAATCGTGATTA
CAGAAAAAAAACAGGGCGCTGGAAAAGTGAAGAATCCGAAATTTTTTTCGAAATCACCATTGTTTGTTTTGAGTAGA
TCAAAGTCTTGAAAGGTGCAGCAAGATATAGGATCTTGACCTGAAGAGTATTGATAACGAACTACATCACATATTGT
ATCAAATA**ATGGTGAGCAAGGGCGAGGAGGATAACATGGCCATCATCAAGGAGTTCATGCGCTTCAAGGTGCACATG**
GAGGGCTCCGTGAACGGCCACGAGTTCGAGATCGAGGGCGAGGGCGAGGGCCGCCCCTACGAGGGCACCCAGACCGC
CAAGCTGAAGGTGACCAAGGGTGGCCCCCTGCCCTTCGCCTGGGACATCCTGTCCCCTCAGTTCATGTACGGCTCCA
AGGCCTACGTGAAGCACCCCGCCGACATCCCCGACTACTTGAAGCTGTCCTTCCCCGAGGGCTTCAAGTGGGAGCGC
GTGATGAACTTCGAGGACGGCGGCGTGGTGACCGTGACCCAGGACTCCTCCCTGCAGGACGGCGAGTTCATCTACAA
GGTGAAGCTGCGCGGCACCAACTTCCCCTCCGACGGCCCCGTAATGCAGAAGAAGACCATGGGCTGGGAGGCCTCCT
CCGAGCGGATGTACCCCGAGGACGGCGCCCTGAAGGGCGAGATCAAGCAGAGGCTGAAGCTGAAGGACGGCGGCCAC
TACGACGCTGAGGTCAAGACCACCTACAAGGCCAAGAAGCCCGTGCAGCTGCCCGGCGCCTACAACGTCAACATCAA
GTTGGACATCACCTCCCACAACGAGGACTACACCATCGTGGAACAGTACGAACGCGCCGAGGGCCGCCACTCCACCG
GCGGCATGGACGAGCTGTACAAGTAAGTTGTATCTGTACAAAATCCAAAGCTGAGCAAATAAATAAATAAATAAATG
TATAAGTTACCGAACGGGGGTATTTTTACTTTTGATCAAAAATTTATGTACCAACTACAAAGTTTCCTCAGCACAGC
CTTCAAGAAGGGAACACACATACAAACAGTGTCAAATAATTGTAGGGATAAATTTAAATATGGCATAAACTAAATAA
GTAGAGCATGAAAAAACTGCAAAATCCAAAAAGTAAAAACGAAGGTCAGAAAGTAAAGCAAAAGAAAATTAATAAAG
CAATACTAAATCTATCATGATTTCCCGTAACTTCCATTAAAGCTGTAACCAGATTTACTCCTACTGTTTGAGCCTCT
AACGCCTAATGGATTTTTAGAGAAGCTCAACCTGATACCTCCTTTGTTGTTGAGGGAAGGGCGGGGGTGAGGTAGTT
GACTACCATATAATTCTGCCAATGCTCTAGTGGCAAAGCTAACATCCTCACAAAGCAAAATAAAAGAAACTTAATAC
GTTATGCCGTAATGAAGGGCTACCAAAAACGATAATCTCAACTGTAAACAGGTACAATGCGGACCCTTTTGCCACAA
AACATACATCATTCATTGCCGGAAAAAGAAAGAAGTGAAGACAGCAGTGCAGCCAGCCATGTTGCGCCAATCTAATT
ATAGATGCTGGTGCCCTGAGGATGTATCTGGAGCCAGCCATGGCATCATGCGCTACCGCCGGATGTAAAATCCGACA
CGCAAAAGAAAACCTTCGAGGTTGCGCACTTCGCCCACCCATGAACCACACGGTTAGTCCAAAAGGGGCAGTTCAGA
TTCCAGATGCGGGAATTAGCTTGCTGCCACCCTCACCTCACTAACGCTGCGGTGTGCGGATACTTCATGCTATTTAT
AGACGCGCGTGTCGGAATCAGCACGCGCAAGAACCAAATGGGAAAATCGGAATGGGTCCAGAACTGCTTTGAGTGCT
GGCTATTGGCGTCTGATTTCCGTTTTGGGAATCCTTTGCCGCGCGCCCCTCTCAAAACTCCGCACAAGTCCCAGAAA
GCGGGAAAGAAATAAAACGCCACCAAAAAAAAAAAAAATAAAAGCCAATCCTCGAAGCGTGGGTGGTAGGCCCTGGAT
TATCCCGTACAAGTATTTCTCAGGAGTAAAAAAACCGTTTGTTTTGGAATTCCCCATTTCGCGGCCACCTACGCCGC
TATCTTTGCAACAACTATCTGCGATAACTCAGCAAATTTTGCATATTCGTGTTGCAGTATTGCGATAATGGGAGTCT
TACTTCCAACATAACGGCAGAAAGAAATGTGAGAAAATTTTGCATCCTTTGCCTCCGTTCAAGTATATAAAGTCGGC
ATGCTTGATAATCTTTCTTTCCATCCTACATTGTTCTAATTATTCTTATTCTCCTTTATTCTTTCCTAACATACCAA
GAAATTAATCTTCTGTCATTCGCTTAAACACTATATCAATA**ATGAGGAGGGCTTTTGTAGAAAGAAATACGAACGAA**
ACGAAAATCAGCGTTGCCATCGCTTTGGACAAAGCTCCCTTACCTGAAGAGTCGAATTTTATTGATGAACTTATAAC
TTCCAAGCATACAAACCAAAAGGGAGAACAAGTAATCCAAGTAGACACGGGAATTGGATTCTTGGATCACATGTATC
ATGCACTGGCTAAACATGCAGGCTGGAGCTTACGACTTTACTCAAGAGGTGATTTAATCATCGATGATCATCACACT
GCAGAAGATACTGCTATTGCACTTGGTATTGCATTCAAGCAGGCTATGAGTAACTTTGCCGGCGTTAAAAGATTTGG
ACATGCTTATTGTCCACTTGACGAAGCTCTTTCTAGAAGCGTAGTTGACTTGTCGGGACGGCCCTATGCTGTTATCG
ATTTGGGATTAAAGCGTGAAAAGGTTGGGGAATTGTCCTGTGAAATGATCCCTCACTTACTATATTCCTTTTCGGTA
GCAGCTGGAATTACTTTGCATGTTACCTGCTTATATGGTAGTAATGACCATCATCGTGCTGAAAGCGCTTTTAAATC
TCTGGCTGTTGCCATGCGCGCGGCTACTAGTCTTACTGGAAGTTCTGAAGTCCCAAGCACGAAGGGAGTGTTGTAAA
GAGTACTGACAATAAAAAGATTCTTGTTTTCAAGAACTTGTCATTTGTATAGTTTTTTTATATTGTAGTTGTTCTAT
TTTAATCAAATGTTAGCGTGATTTATATTTTTTTTCGCCTCGACATCATCTGCCCAGATGCGAAGTTAAGTGCGCAG
AAAGTAATATCATGCGTCAATCGTATGTGAATGCTGGTCGCTATACTGCTGTCGATTCGATACTAACGCCGCCATCC
AGTTTTCCGGTGGACTTCGGCTACGTAGGGAGTGACACCGATTATTTAAAGCTGCAGCATACGATATATATACATGT
GTATATATGTATACCTATGAATGTCAGTAAGTATGTATACGAACAGTATGATACTGAAGATGACAAGGTAATGCATC
ATTCTATACGTGTCATTCTGAACGAGGCGCGCTTTCCTTTTTTCTTTTTGCTTTTTCTTTTTTTTTCTCTTGAACTC
GAGAAAAAAAATATAAAAGAGATGGAGGAACGGGAAAAAGTTAGTTGTGGTGATAGGTGGCAAGTGGTATTCCGTAA
GAACAACAAGAAAAGCATTTCATATTATGGCTGAACTGAGCGAACAAGTGCAAAATTTAAGCATCAACGACAACAAC
GAGAATGGTTATGTTCCTCCTCACTTAAGAGGAAAACCAAGAAGTGCCAGAAATAACAGTAGCAACTACAATAACAA
CAACGGCGGCTACAACGGTGGCCGTGGCGGTGGCAGCTTCTTTAGCAACAACCGTCGTGGTGGTTACGGCAACGGTG
GTTTCTTCGGTGGAAACAACGGTGGCAGCAGATCTAACGGCCGTTCTGGTGGTAGATGGATCGATGGCAAACATGTC
CCAGCTCCAAGAAACGAAAAGGCCGAGATCGCCATATTTGGTGTCCCCGAGGATCCAAATTTCCAATCTTCTGGTAT
TAACTTCGATAACTACGATGATATTCCAGTGGACGCCTCTGGTAAGGATGTTCCTGAACCAATCACAGAATTTACCT
CACCTCCATTGGACGGATTGTTATTGGAAAACATCAAATTGGCCCGTTTCACCAAGCCAACACCTGTGCAAAAATAC
TCCGTCCCTATCGTTGCCAACGGCAGAGATTTGATGGCCTGTGCGCAGACCGGTTCTGGTAAGACTGGTGGGTTTTT
ATTCCCAGTGTTGTCCGAATCATTTAAGACTGGACCATCTCCTCAACCAGAGTCTCAAGGCTCCTTTTACCAAAGAA
AGGCCTACCCAACTGCTGTC

sgRNAs Expression Cassettes

prSNR52::***crRNA***::*tracrRNA*::***SUP4(t)***

pRS425 + sgRNA(u1) (Vector name, pGF-V1220)

GGATCCTCACTAAAGGGAACAAAAGCTGGAGCTTCTTTGAAAAGATAATGTATGATTATGCTTTCACTCATATTTAT
ACAGAAACTTGATGTTTTCTTTCGAGTATATACAAGGTGATTACATGTACGTTTGAAGTACAACTCTAGATTTTGTA
GTGCCCTCTTGGGCTAGCGGTAAAGGTGCGCATTTTTTCACACCCTACAATGTTCTGTTCAAAAGATTTTGGTCAAA
CGCTGTAGAAGTGAAAGTTGGTGCGCATGTTTCGGCGTTCGAAACTTCTCCGCAGTGAAAGATAAATGATC**CGGTGG**
ACTTCGGCTACGTAGTTTTAGAGCTAGAAATAGCAAGTTAAAATAAGGCTAGTCCGTTATCAACTTGAAAAAGTGGC
ACCGAGTCGGTGGTGC**TTTTTTTGTTTTTTATGTCT**TCGAGTCATGTAATTAGTTATGTCACGC**CTCGAG**

pRS425 + sgRNA(u2) (Vector name, pGF-V809)

GGATCCTCACTAAAGGGAACAAAAGCTGGAGCTTCTTTGAAAAGATAATGTATGATTATGCTTTCACTCATATTTAT
ACAGAAACTTGATGTTTTCTTTCGAGTATATACAAGGTGATTACATGTACGTTTGAAGTACAACTCTAGATTTTGTA
GTGCCCTCTTGGGCTAGCGGTAAAGGTGCGCATTTTTTCACACCCTACAATGTTCTGTTCAAAAGATTTTGGTCAAA
CGCTGTAGAAGTGAAAGTTGGTGCGCATGTTTCGGCGTTCGAAACTTCTCCGCAGTGAAAGATAAATGATC**GCTGTT**
CGTGTGCGCGTCCTGTTTTAGAGCTAGAAATAGCAAGTTAAAATAAGGCTAGTCCGTTATCAACTTGAAAAAGTGGC
ACCGAGTCGGTGGTGC**TTTTTTTGTTTTTTATGTCT**TCGAGTCATGTAATTAGTTATGTCACGC**CTCGAG**

pRS425 + sgRNA(*SpHIS5*) (Vector name, pGF-V2152)
pRS426 + sgRNA(*SpHIS5*) (Vector name, pGF-V2153)

GCGGCCGCTCACTAAAGGGAACAAAAGCTGGAGCTTCTTTGAAAAGATAATGTATGATTATGCTTTCACTCATATTT
ATACAGAAACTTGATGTTTTCTTTCGAGTATATACAAGGTGATTACATGTACGTTTGAAGTACAACTCTAGATTTTG
TAGTGCCCTCTTGGGCTAGCGGTAAAGGTGCGCATTTTTTCACACCCTACAATGTTCTGTTCAAAAGATTTTGGTCA
AACGCTGTAGAAGTGAAAGTTGGTGCGCATGTTTCGGCGTTCGAAACTTCTCCGCAGTGAAAGATAAATGATC**ACAA**
GTAATCCAAGTAGACAGTTTTAGAGCTAGAAATAGCAAGTTAAAATAAGGCTAGTCCGTTATCAACTTGAAAAAGTG
GCACCGAGTCGGTGGTGC**TTTTTTTGTTTTTTATGTCT**TCGAGTCATGTAATTAGTTATGTAAC**ACTAGT**

pRS425 + sgRNA(mCherry) (Vector name, pGF-V2158)
pRS426 + sgRNA(mCherry) (Vector name, pGF-V2159)

GGATCCTCACTAAAGGGAACAAAAGCTGGAGCTTCTTTGAAAAGATAATGTATGATTATGCTTTCACTCATATTTAT
ACAGAAACTTGATGTTTTCTTTCGAGTATATACAAGGTGATTACATGTACGTTTGAAGTACAACTCTAGATTTTGTA
GTGCCCTCTTGGGCTAGCGGTAAAGGTGCGCATTTTTTCACACCCTACAATGTTCTGTTCAAAAGATTTTGGTCAAA
CGCTGTAGAAGTGAAAGTTGGTGCGCATGTTTCGGCGTTCGAAACTTCTCCGCAGTGAAAGATAAATGATC**CAAGGA**
GTTCATGCGCTTCAGTTTTAGAGCTAGAAATAGCAAGTTAAAATAAGGCTAGTCCGTTATCAACTTGAAAAAGTGGC
ACCGAGTCGGTGGTGC**TTTTTTTGTTTTTTATGTCT**TCGAGTCATGTAATTAGTTATGTCACGC**CTCGAG**

pRS425 + sgRNA(Kan^R) (Vector name, pGF-V1642)

GGATCCTCACTAAAGGGAACAAAAGCTGGAGCTTCTTTGAAAAGATAATGTATGATTATGCTTTCACTCATATTTAT
ACAGAAACTTGATGTTTTCTTTCGAGTATATACAAGGTGATTACATGTACGTTTGAAGTACAACTCTAGATTTTGTA
GTGCCCTCTTGGGCTAGCGGTAAAGGTGCGCATTTTTTCACACCCTACAATGTTCTGTTCAAAAGATTTTGGTCAAA
CGCTGTAGAAGTGAAAGTTGGTGCGCATGTTTCGGCGTTCGAAACTTCTCCGCAGTGAAAGATAAATGATC**GCCATC**
CTATGGAACTGCCTGTTTTAGAGCTAGAAATAGCAAGTTAAAATAAGGCTAGTCCGTTATCAACTTGAAAAAGTGGC
ACCGAGTCGGTGGTGC**TTTTTTTGTTTTTTATGTCT**TCGAGTCATGTAATTAGTTATGTCACGC**CTCGAG**

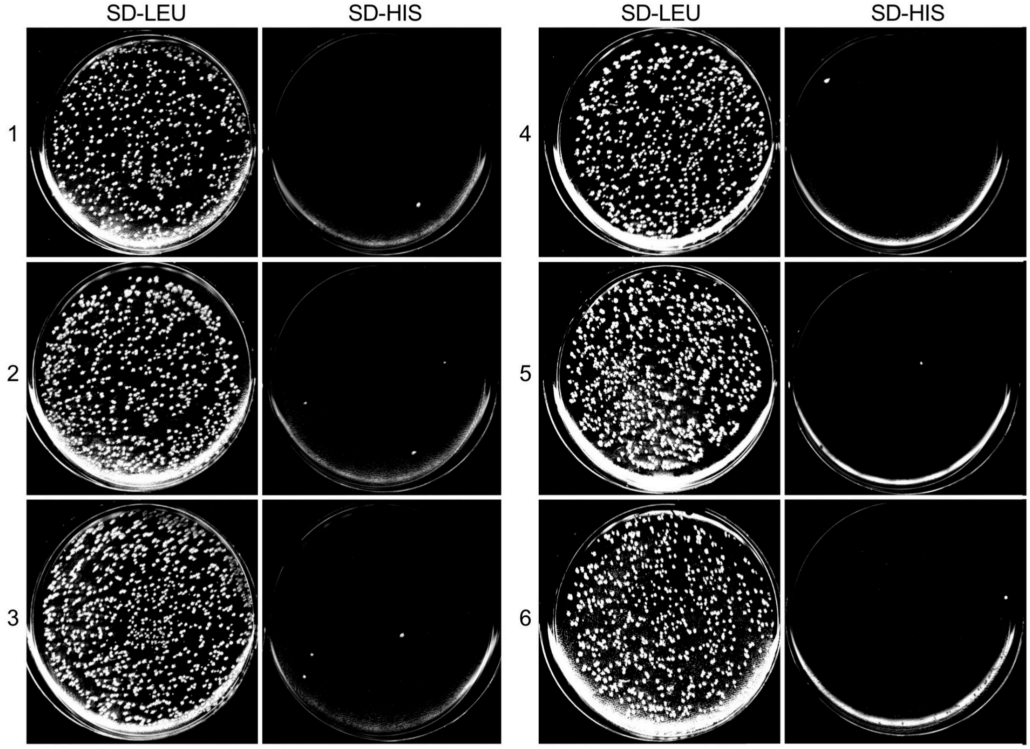

Figure S2. Gene drive growth assays for first-generation (GD1) system in budding yeast. From Fig. 1C, additional examples of diploid gene drive strains tested on SD-LEU (control plate to select for sgRNA(u1)-containing plasmid) and SD-HIS (experimental condition to assay for the presence of the *S. pombe HIS5* gene within the target allele). GD1 diploid strains were generated through independent mating of the parental haploid drive (GFY-2383) and target (GFY-3733) yeast and subsequent diploid selection steps. All examples shown (as well as multiple repetitions) display a near 100% drive activity with few surviving colonies on the SD-HIS condition. Images of individual plates were edited for contrast (each plate separately). Horizontal and vertical white lines denote separate plates.

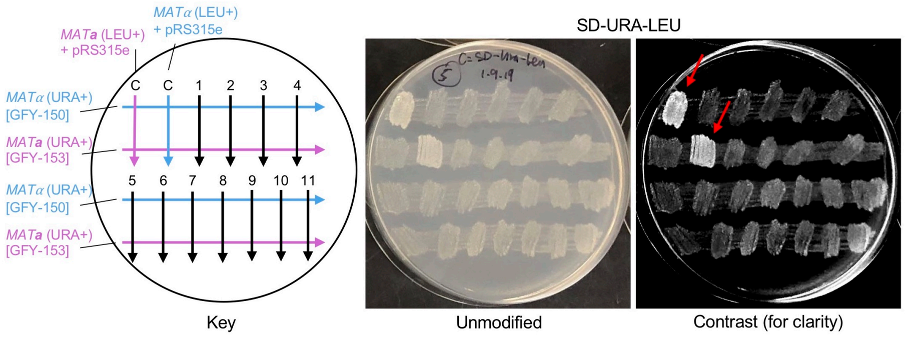

Figure S3. Determination of ploidy status through a mating and diploid selection assay. Following gene drive activation, strains were selected as clonal isolates on permissive media (for example, SD-LEU) and tested against known haploid control strains (including GFY-150/GFY-153 or BY4742/BY4741). Haploids were mated on YPD plates for 24 h and then transferred by sterile velvets to selection plates for 24 h prior to imaging. Selection plates (such as SD-URA-LEU) provided a growth challenge where only diploid strains harboring both selection markers (one from each haploid strain) would survive. The unmodified and edited (for contrast) plates shown are identical. For this example, control strains (GFY-150 and GFY-153) harbored a *URA3*-based *CEN* plasmid. The haploid positive controls (labeled "C") included BY4741 and BY4742 containing an empty *CEN*-based pRS315 plasmid (marked with *LEU2*). The clonal samples (labeled 1-11) selected after activation of the gene drive assay (Fig. 1) all contained a *LEU2*-based plasmid (harboring the sgRNA) but no *URA3*-based marker. For control combinations of a *MAT**a*** mated to a *MATα* strain, diploids were able to survive on media testing for the presence of both *LEU2*+ and *URA3*+ plasmids (red arrows). The GD1 diploid yeast, unable to mate to either of the *URA3*-containing strains, were unable to grow on this selection plate. This ploidy growth test was also repeated in an independent assay using the pRS313-containing WT strains (*HIS3*) to mate against the clonal diploids from the gene drive assay (that had lost *SpHIS5*) with a similar procedure (selection on SD-HIS-LEU plates).

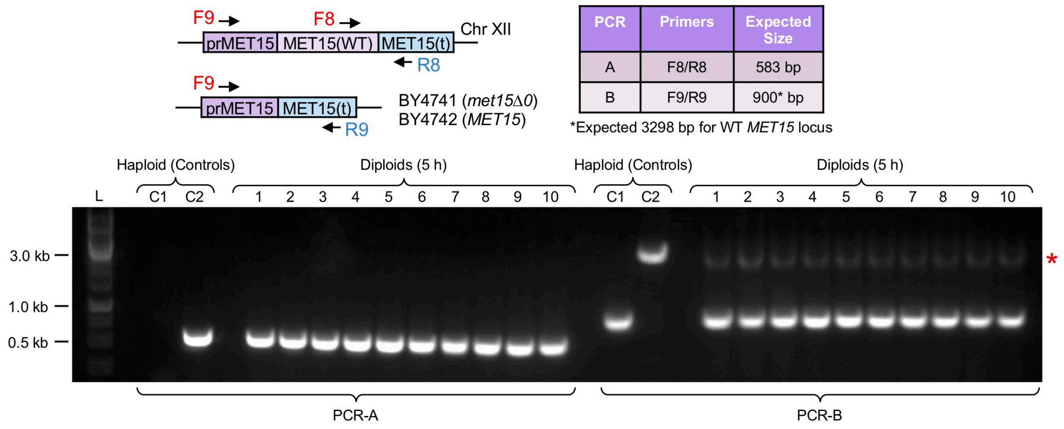

Figure S4. PCR amplification of the *MET15* locus to assay ploidy status. As an independent test (compared to *LYS2*) of whether yeast strains following gene drive activation were haploid or diploid, we examined the *MET15* (also termed *MET17* or *MET25*) locus on chromosome XII. BY4741 haploid yeast are *met15Δ0* whereas BY4742 haploid yeast are *MET15*. We included the two haploid parental strains harboring the drive (GFY-2383) and target constructs (GFY-3733) at the *HIS3* locus (labeled "C1" and "C2," respectively). We chose ten isolates (1-10) from the gene drive assay (Fig. 1) that were tested as diploids using the mating test (described in Fig. S3). One set of oligonucleotides (PCR A) tested for the presence of the *MET15* coding sequence. The second set (PCR B) amplified the entire *MET15* locus from within the promoter and terminator regions. For the first PCR (*left*), the BY4742 (target) haploid and all 10 isolates displayed a fragment at the expected size of 583 bp. For the second PCR, two fragment sizes were expected: (i) *met15Δ0* would yield a product size of 900 bp whereas the *MET15* locus would yield a band of 3,298 bp. These amplified fragments were seen for haploid controls. However, for the GD1 isolates, two bands were observed at both sizes (red asterisk marks the larger band). Note, PCR reactions were optimized for generation of the 900 bp fragment. These data support that these gene drive strains included both the *met15Δ0* and *MET15* alleles and were diploid.

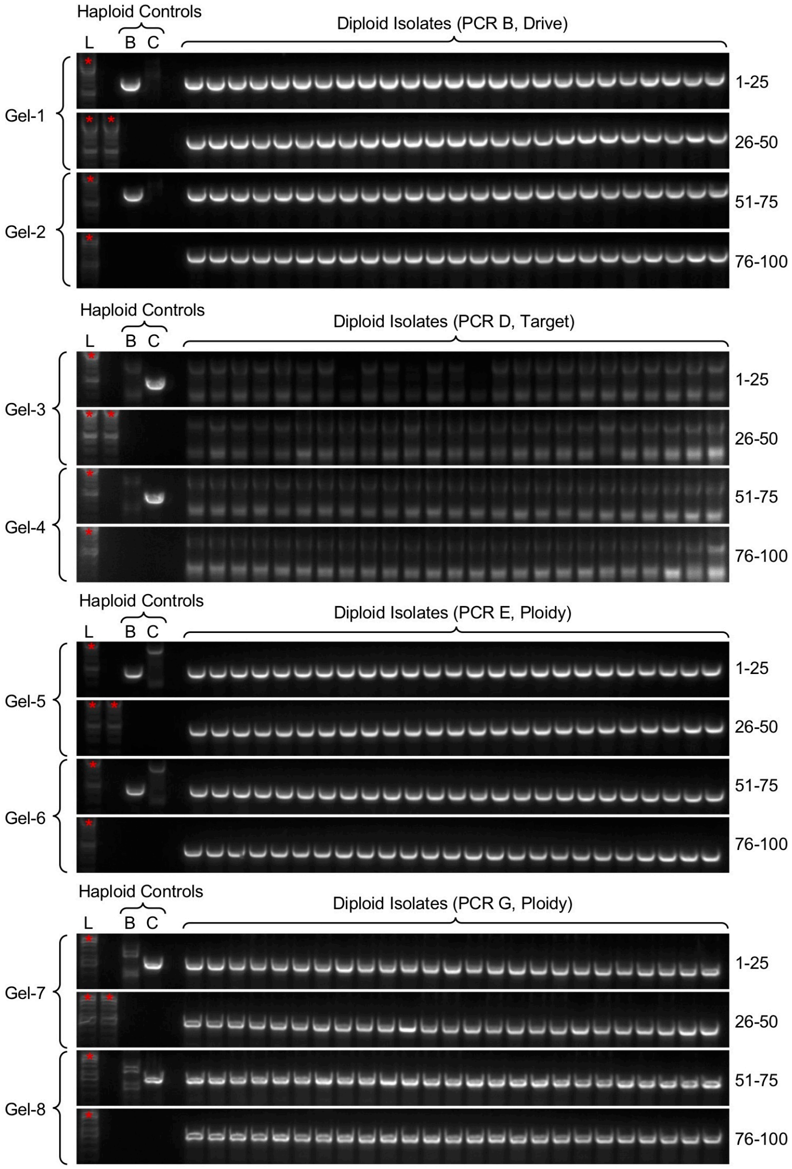

Figure S5. PCR amplification of the *HIS3* and *LYS2* loci for 100 separate gene drive diploid isolates. Samples 1-100 correspond to the same clones from Fig. 2C (isolated after 5 h activation of the drive from SD-LEU plates). Note, these 100 samples also include the 20 isolates displayed in Fig. 1D (20 + 80 new isolates); the PCRs have been repeated with the entire set of 100 chromosomal DNA preparations using similar conditions. The drive allele was confirmed (PCR B) using oligonucleotides F2/R2 (PCRs include identical labels from Fig. 1D). The target allele was amplified (PCR D) using primers F4/R4. The *LYS2* locus was amplified (PCRs E,G) using

primers F5/R5 and F7/R7, respectively. Red asterisks, positions of included DNA ladders (“L”). Two haploid controls (labeled “B” and “C”) are the original drive haploid strain (GFY-2383) and target haploid strain (GFY-3733), as in Fig. 1D. White lines indicate separate DNA gels. Note, the first and second gels were run together on a single agarose gel (two separate rows); the third and fourth gels were run in a similar fashion. One set of haploid controls was run per complete gel (for example, the first and third gels). No additional image processing or editing (aside from cropping for clarity) has been done. The isolate number is included on the far right of each image. These data demonstrate these 100 isolates were diploids, contained the drive allele, and had lost the target allele.

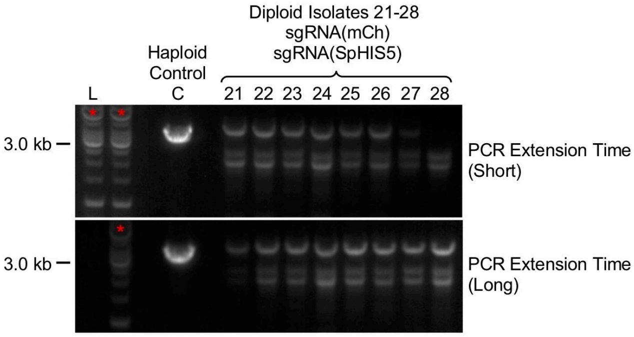

PCR	Primers	Expected Size
Target	F12/R4	3531* bp

Figure S6. PCR amplification of the target allele for gene drive isolates harboring two guide RNAs to target mCherry and *S. pombe HIS5*. NHEJ-based repair did not occur for any examined isolates (21-28) from Fig. 3C. PCRs were performed on GD1 diploid isolates following drive activation; the haploid target strain (GFY-3733) served as a control ("C"). The expected product size for the full-length target allele was 3,531 bp. Assuming dual cleavage at both the mCherry and *SpHIS5* sites and exacting repair via NHEJ, the expected PCR fragment size would be 1,240 bp. For all tested isolates (21-28), neither band was observed, despite two independent PCR reactions using extension times optimized for either the shorter fragment (*top*) or longer fragment (*bottom*). Red asterisks, DNA ladders ("L"). Gel images have not been processed aside from cropping for clarity.

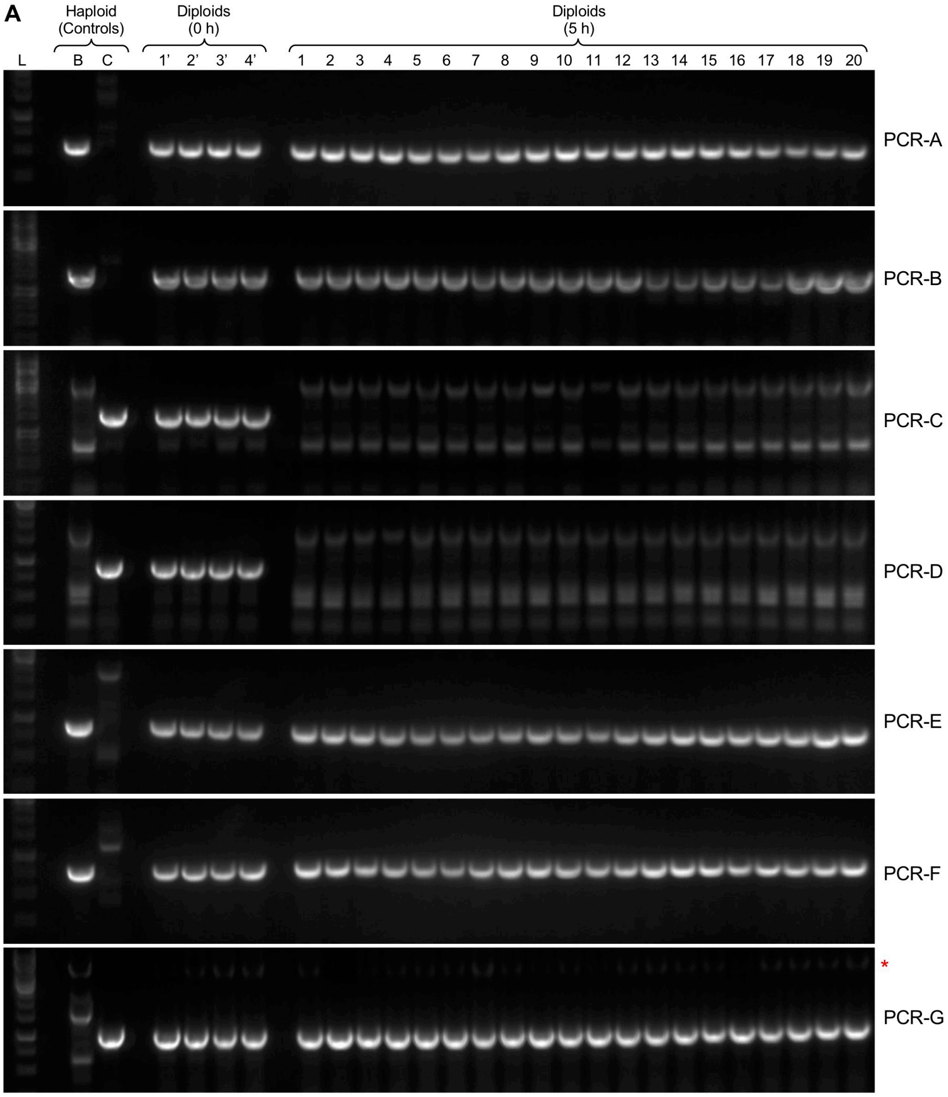

Figure S7. Original DNA agarose gel images used to visualize PCRs from Figs. 1-5. All images shown were obtained using an Invitrogen E-Gel™ Imager (ThermoFisher Scientific) and were cropped for positioning and clarity, but were not altered by other methods. DNA molecular ladders (labeled "L"). Red asterisk, faint PCR band (5,573 bp) corresponding to the WT *LYS2* locus. Horizontal and vertical white lines denote separate DNA gels. (A) Gels from Fig. 1. (B) Gels from Fig. 2. In some gels, a band for Control-C can be seen at approximately 3,930 bp. (C) Gels from Fig. 3. (D) Gels from Fig. 4. (E) Gels from Fig. 5. Double red asterisk, no ladder was included within nearby lanes as this image was cropped from a larger gel. Figures S7, B-E are found below.

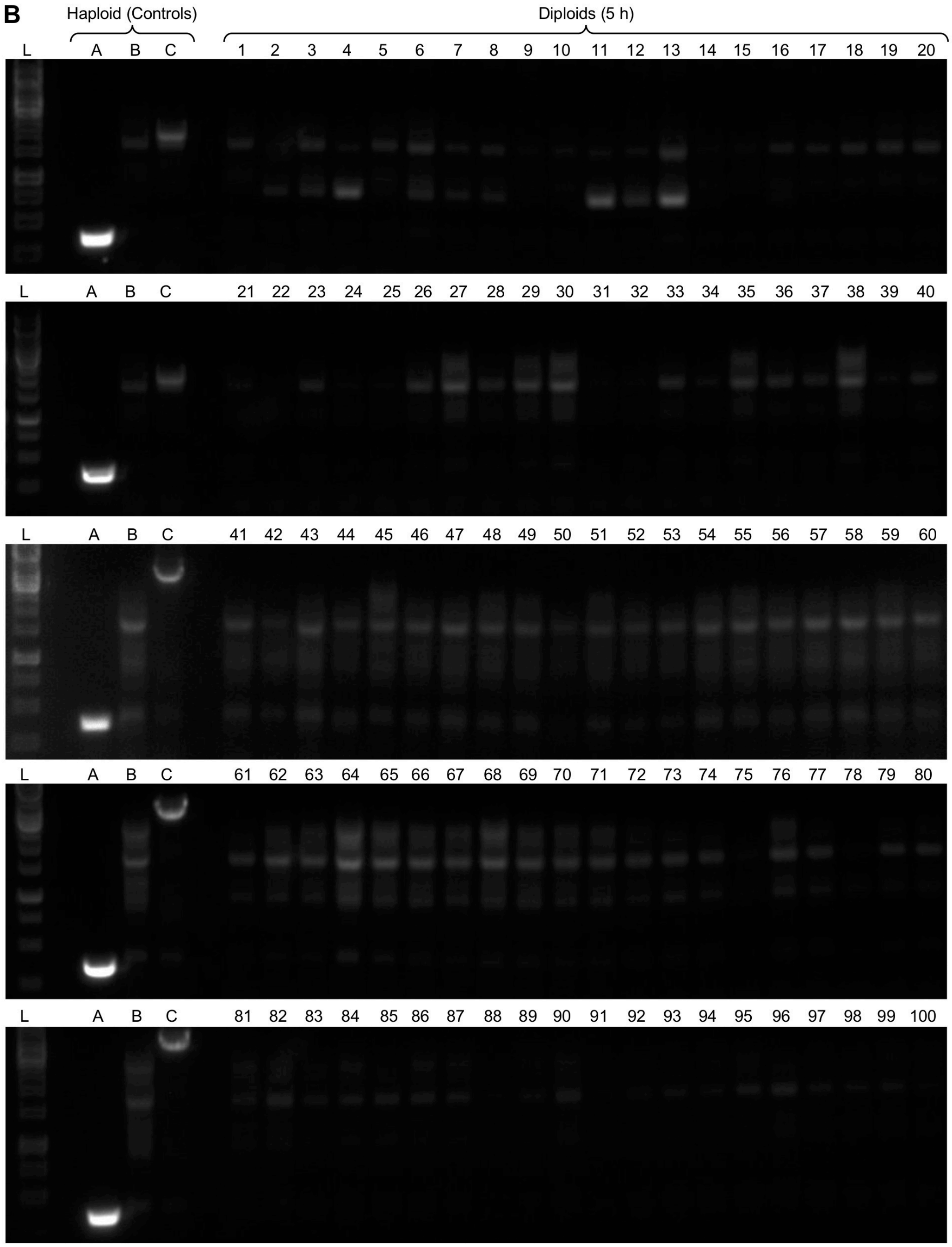
B
Haploid (Controls)
Diploids (5 h)
L A B C 1 2 3 4 5 6 7 8 9 10 11 12 13 14 15 16 17 18 19 20
L A B C 21 22 23 24 25 26 27 28 29 30 31 32 33 34 35 36 37 38 39 40
L A B C 41 42 43 44 45 46 47 48 49 50 51 52 53 54 55 56 57 58 59 60
L A B C 61 62 63 64 65 66 67 68 69 70 71 72 73 74 75 76 77 78 79 80
L A B C 81 82 83 84 85 86 87 88 89 90 91 92 93 94 95 96 97 98 99 100

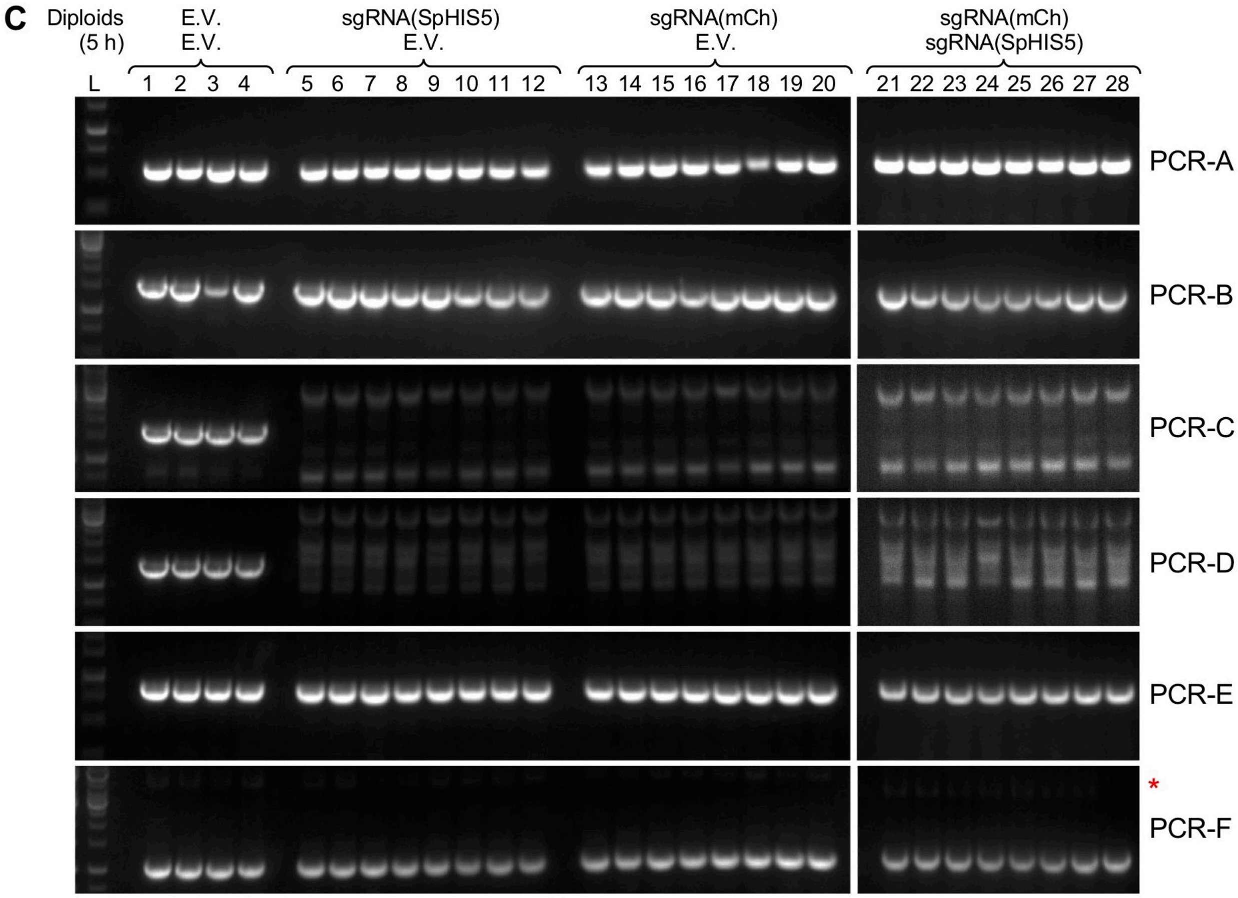

C
Diploids (5 h)
E.V.
E.V.
sgRNA(SpHIS5)
E.V.
sgRNA(mCh)
E.V.
sgRNA(mCh)
sgRNA(SpHIS5)
L 1 2 3 4 5 6 7 8 9 10 11 12 13 14 15 16 17 18 19 20 21 22 23 24 25 26 27 28
PCR-A
PCR-B
PCR-C
PCR-D
PCR-E
*
PCR-F

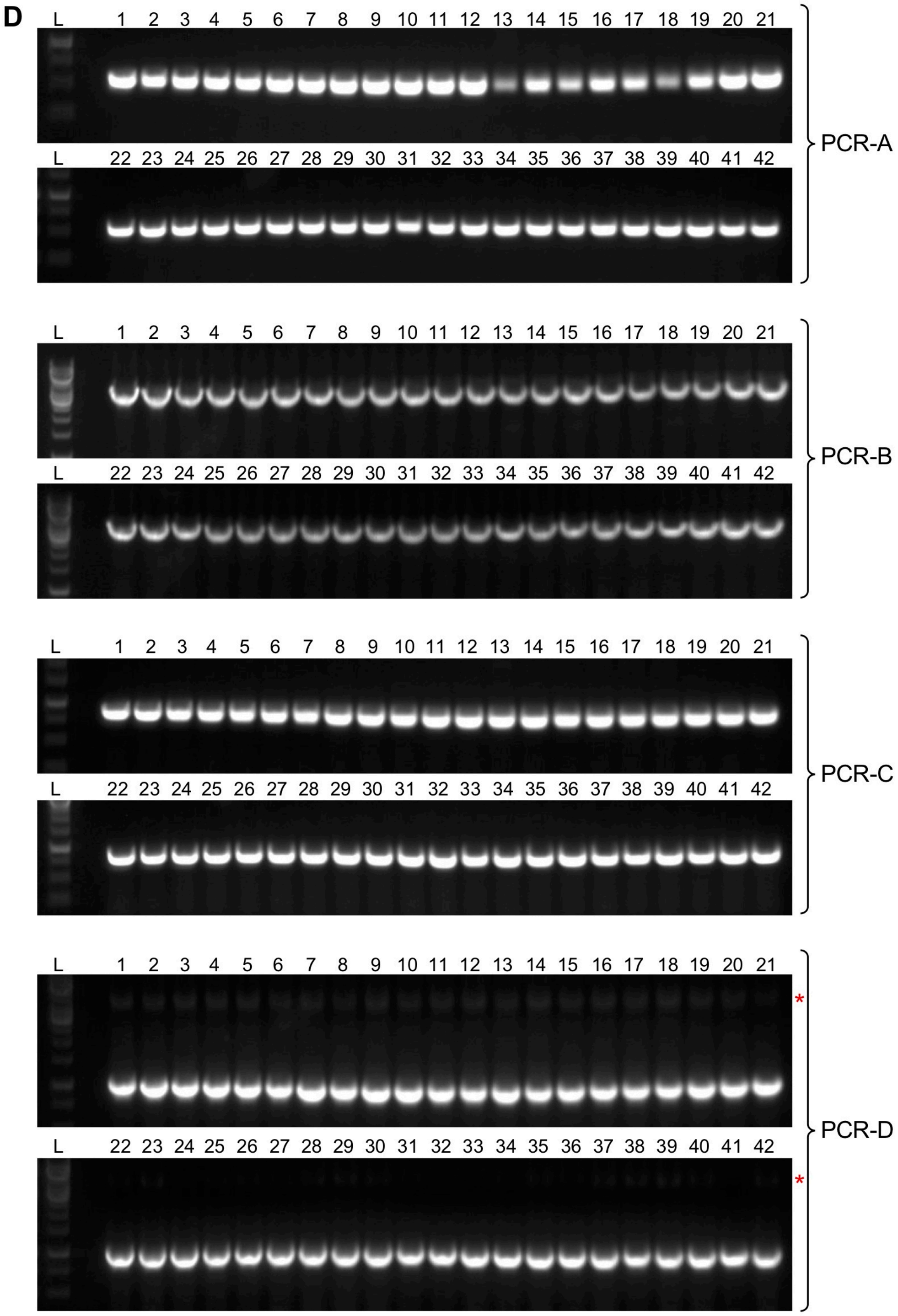
D
L 1 2 3 4 5 6 7 8 9 10 11 12 13 14 15 16 17 18 19 20 21
L 22 23 24 25 26 27 28 29 30 31 32 33 34 35 36 37 38 39 40 41 42
PCR-A
PCR-B
PCR-C
PCR-D
*

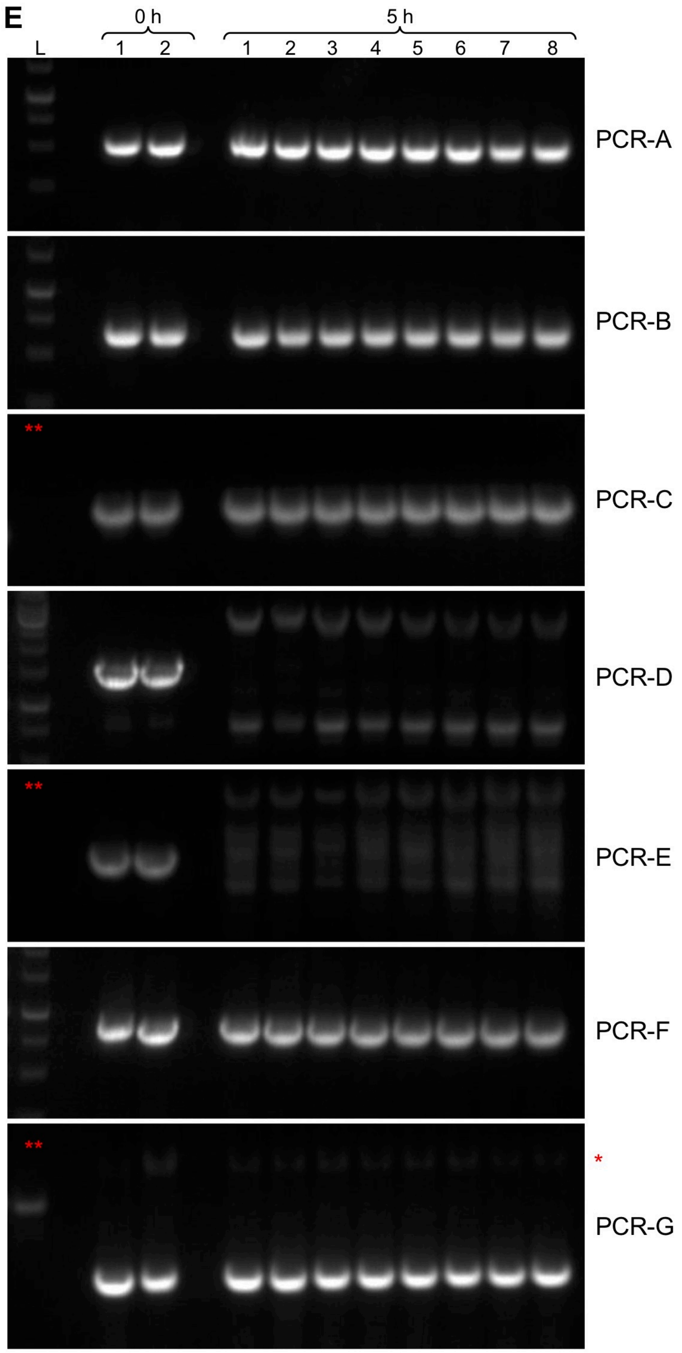
E
0 h
5 h
L
1
2
1
2
3
4
5
6
7
8
PCR-A
PCR-B
**
PCR-C
PCR-D
**
PCR-E
PCR-F
**
*
PCR-G

REFERENCES

1 Brachmann, C. B. *et al.* Designer deletion strains derived from *Saccharomyces cerevisiae* S288C: a useful set of strains and plasmids for PCR-mediated gene disruption and other applications. *Yeast (Chichester, England)* **14**, 115-132, doi:10.1002/(sici)1097-0061(19980130)14:2<115::aid-yea204>3.0.co;2-2 (1998).

2 Roggenkamp, E. *et al.* Tuning CRISPR-Cas9 Gene Drives in *Saccharomyces cerevisiae*. *G3 (Bethesda, Md.)* **8**, 999-1018, doi:10.1534/g3.117.300557 (2018).

3 Finnigan, G. C., Takagi, J., Cho, C. & Thorner, J. Comprehensive Genetic Analysis of Paralogous Terminal Septin Subunits Shs1 and Cdc11 in *Saccharomyces cerevisiae*. *Genetics* **200**, 821-841, doi:10.1534/genetics.115.176495 (2015).

4 Versele, M. *et al.* Protein-protein interactions governing septin heteropentamer assembly and septin filament organization in *Saccharomyces cerevisiae*. *Molecular biology of the cell* **15**, 4568-4583, doi:10.1091/mbc.E04-04-0330 (2004).

5 Finnigan, G. C. & Thorner, J. mCAL: a new approach for versatile multiplex action of Cas9 using one sgRNA and loci flanked by a programmed target sequence. *G3 (Bethesda, Md.)* **6**, 2147-2156, doi:10.1534/g3.116.029801 (2016).

6 Christianson, T. W., Sikorski, R. S., Dante, M., Shero, J. H. & Hieter, P. Multifunctional yeast high-copy-number shuttle vectors. *Gene* **110**, 119-122 (1992).

7 Sikorski, R. S. & Hieter, P. A system of shuttle vectors and yeast host strains designed for efficient manipulation of DNA in *Saccharomyces cerevisiae*. *Genetics* **122**, 19-27 (1989).

8 Roggenkamp, E. *et al.* CRISPR-UnLOCK: multipurpose Cas9-based strategies for Conversion of yeast libraries and strains. *Frontiers in microbiology* **8**, 1773, doi:10.3389/fmicb.2017.01773 (2017).

9 DiCarlo, J. E. *et al.* Genome engineering in *Saccharomyces cerevisiae* using CRISPR-Cas systems. *Nucleic acids research* **41**, 4336-4343, doi:10.1093/nar/gkt135 (2013).

10 Finnigan, G. C. & Thorner, J. Complex *in vivo* ligation using homologous recombination and high-efficiency plasmid rescue from *Saccharomyces cerevisiae*. *Bio-protocol* **5**, e1521. http://www.bio-protocol.org/e1521 (2015).
